# Early Warning Signals of Social Transformation: A Case Study from the US Southwest

**DOI:** 10.1371/journal.pone.0163685

**Published:** 2016-10-05

**Authors:** Katherine A. Spielmann, Matthew A. Peeples, Donna M. Glowacki, Andrew Dugmore

**Affiliations:** 1 School of Human Evolution and Social Change, Arizona State University, Tempe, Arizona, United States of America; 2 Department of Anthropology, University of Notre Dame, Notre Dame, Indiana, United States of America; 3 Institute of Geography and the Lived Environment, School of Geosciences, University of Edinburgh, Edinburgh, Scotland, United Kingdom; University College Dublin, IRELAND

## Abstract

Recent research in ecology suggests that generic indicators, referred to as *early warning signals* (EWS), may occur before significant transformations, both critical and non-critical, in complex systems. Up to this point, research on EWS has largely focused on simple models and controlled experiments in ecology and climate science. When humans are considered in these arenas they are invariably seen as external sources of disturbance or management. In this article we explore ways to include societal components of socio-ecological systems directly in EWS analysis. Given the growing archaeological literature on ‘collapses,’ or transformations, in social systems, we investigate whether any early warning signals are apparent in the archaeological records of the build-up to two contemporaneous cases of social transformation in the prehistoric US Southwest, Mesa Verde and Zuni. The social transformations in these two cases differ in scope and severity, thus allowing us to explore the contexts under which warning signals may (or may not) emerge. In both cases our results show increasing variance in settlement size before the transformation, but increasing variance in social institutions only before the critical transformation in Mesa Verde. In the Zuni case, social institutions appear to have managed the process of significant social change. We conclude that variance is of broad relevance in anticipating social change, and the capacity of social institutions to mitigate transformation is critical to consider in EWS research on socio-ecological systems.

## Introduction

**Fig 1:**
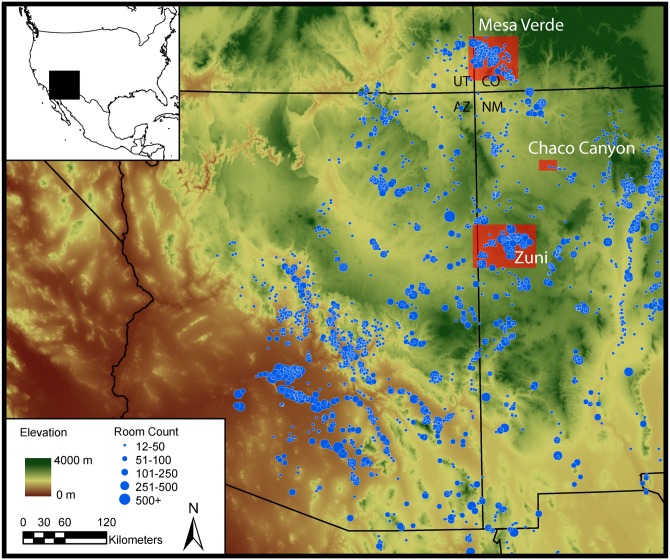
Map of the U.S. Southwest. The map shows the late pre-Hispanic distribution of major settlements ca. 1250–1300 C.E., and the locations of the Zuni and Mesa Verde areas and of Chaco Canyon.

It is difficult to predict when complex systems are approaching thresholds where a small change (forcing) can result in a significant transformation. Recent research in ecology suggests that generic indicators, referred to as *early warning signals*, may occur before such transformations. Although Scheffer and co-authors [1] have suggested that the social sciences are an important arena in which to expand early warning signals analysis, such analyses are rarely undertaken. Indeed, within both modelling and empirical studies of socio-ecological systems (SES) humans are invariably seen as external sources of disturbance or management. Our goal is to explore ways to include societal components of SES within the systems under investigation. Given the growing archaeological literature on ‘collapses’ in social systems (e.g., [2,3,4,5]), and our long-term participation in collaborative and comparative research concerning social transformation in the US Southwest and the North Atlantic [6], we undertook a series of analyses to determine whether early warning signals are apparent in the archaeological records of the build-up to two contemporaneous cases of social transformation in the pre-Hispanic US Southwest. As numerous researchers have noted (e.g., [7] and references therein), the well-researched and highly detailed paleoclimatic and archaeological records of the US Southwest make it a particularly appropriate region in which to investigate SES and, by implication, to search for possible early warning signals in cultural data. Our two case studies, Mesa Verde and Zuni (Fig 1), differ significantly in the nature and degree of social transformation, thus allowing us to explore the contexts under which warning signals may (or may not) emerge.

Transformation in socio-ecological systems involves reorganization to a distinctly different state. Thus, social transformations entail processes such as notable shifts in the nature and strengths of social relationships, changing community location and structure, institutional change, and changes in identity. Transformations may be either non-critical (reorganization with some continuity) due to resilience within the system [8] or critical (catastrophic), reflecting a regime change to an alternative stable state [9]. Early warning signals reflect the increasing sensitivity of a complex system to external or internal perturbation, and they may occur before both critical [1, 10, 11] and non-critical transformations [12]. In this paper we present analyses that examine the build up to reorganization using data from archaeological research on a non-critical transformation in the Zuni area and a critical transformation in Mesa Verde. We selected these two regions because both form the core of major population concentrations in the late pre-Hispanic period. Each concentration was characterized by strong similarities in material culture and economy indicating frequent interactions at the regional scale. Both cases contain high resolution spatial data on thousands of archaeological sites in closely spaced time steps that can be explored for potential harbingers of social transformation.

In the following we briefly discuss the indicators related to the phenomenon of *critical slowing down* [13] that have been highlighted as signs that a system is close to transformation, and identify increasing variance as the metric most appropriate for our purposes. We then introduce the case studies and discuss our hypotheses related to critical slowing down, our methods of acquiring the variance data, and our results and interpretations. We conclude the paper with a discussion of the strengths of using the “completed experiments” of the past to identify ways that change might be anticipated. We also advocate the explicit inclusion of human dimensions in the study of early warning signals, in what has up to now been largely an ecological examination of socio-ecological systems.

## Key Concepts in Early Warning Signals Analysis

Critical slowing down [1, 9, 11, 14, 15] is a term given to the increased time taken to recover from a small perturbation as a directional driver moves the system towards a critical transition. Drivers can have multiple sources and operate at multiple temporal scales, ranging from slow exogenous systemic changes in a variable, such as climate, to moderate scale changes in endogenous state variables, such as birth and death rates. Researchers have noted the extreme difficulty of measuring recovery rates in real world systems. Instead, it is the temporal and spatial consequences of critical slowing down that are amenable to data collection and analysis. These consequences include increasing autocorrelation and increasing variance and skewness in state variables [9, 16–19]. Temporal autocorrelation increases in the fluctuations between states as recovery slows because the system remains longer in its perturbed state, thus rates of change decrease. Variance is inversely proportional to the return rate of the system and reflects the amplification of small shocks as a dynamic system approaches a critical transition [16, 20].

The settlement data used here include locations and size information for over 2,500 individual habitation structures from over 90,000 acres of full-coverage archaeological survey. These data have a relatively limited number of periods in the time series and contain distributions of values rather than a single metric for our state variables. Therefore we use the coefficient of variation (CoV) to measure variance in system state in order to evaluate whether or not critical slowing down occurred in our case studies prior to social transformation. We did not use other commonly employed analyses of early warning signals in ecological systems because they were either less useful for our purposes or they were inappropriate. For example, autocorrelation is assessed in terms of recovery time, and our data do not have the kinds of signals or sufficient temporal resolution necessary for this evaluation. Skewness is another metric of system instability, but is related to variance [1]. We did evaluate our aggregate site size data in terms of skewness; all time periods for both cases show statistically significant skewness, but, as expected, skewness and CoV are highly correlated (see Table A in S1 File).

Archaeological data provide an enduring record of completed cycles of social transformation, many of which may have relevance for transformations we are experiencing today. Archaeology is thus an ideal domain in which to undertake in-depth comparative analyses of the lead-up to both critical and non-critical social transformations and to assess what factors lead to different outcomes. We recognize that archaeological data differ from the detailed time series ecological data acquired from modeled or extant ecosystems that typify much of the published early warning signals research. Holding all researchers to the same kinds and resolutions of data that ecologists use, however, excludes the important understanding of past human societies that is possible using relevant data available from the archaeological record. Indeed while many environmental records extend over very long time scales, the archaeological record provides the only way to assess key aspects of social change over similarly long time scales. Archaeology is also the only source of direct data on societal change through transformations that occurred beyond the range of oral history or written sources, making it critical to explore ways in which the archaeological record can be used to identify early warning signals. The analyses discussed in this paper illustrate the knowledge that can be gained from data with spatial detail but limited time steps and comparatively coarse temporal resolution. We found the early warning signals approach extremely useful in the comparative analysis of the two case studies introduced below and propose that it be more widely adopted in social science research on transformation.

## The Zuni and Mesa Verde Socio-Ecological Systems

Zuni and Mesa Verde are ideal cases for an early warning signals analysis given the wealth of existing environmental, climatic, and archaeological data from more than a century of research (e.g., [21, 22, 23]). In both regions, the emphasis on archaeological survey and analysis of settlement patterns chronologically anchored by tree-ring dated pottery type distributions allow for inter-regional comparison and an assessment of change over time (e.g., [22, 24, 25]). The period on which we focus, ca. 1020–1375 C.E., includes the lead-up to marked social transformations, which in both cases occurred during the 1200s.

In both cases climate change was a key directional driver over decadal to century timescales, to which people responded in terms of their choice of where and how to live. When people relocated they often created new communities and communal social institutions. In the U.S. Southwest, communal social institutions fused belief, power, and action, rather than separating what today we distinguish as the religious and the political. In the remainder of the paper we thus refer to these communal social institutions as religio-political institutions. Between ca. 1020–1375 C.E., marked periods of drought are one of the overarching external drivers of change across the US Southwest. Although drought is a perennial concern in the US Southwest, this period was complicated by severe and prolonged droughts in the middle 1100s and during the 1200s C.E. [26, 27]. Almost the entire twelfth century, in fact, was dry. While that drought might be considered a sufficient shock in itself to cause a catastrophic shift, neither region experienced radical social or demographic change in the 1100s. Both areas, however, experienced relocation of population to better watered portions of their respective regions in the late 1100s-early 1200s (e.g., [28– 30]). This population relocation became an internal driver of social change, as we discuss below. Although the driver is the same, population relocation led to different outcomes in each region.

### Zuni

The Zuni region is one of only a few places in the Southwestern US that was densely occupied by sedentary small-scale farmers continuously from at least the last millennium B.C.E., through Spanish conquest, and into the present [31, 32]. Our period of interest spans several transformations in Zuni settlement organization and distribution that are clearly recorded in the archaeology of the region. The 11th century was marked by the greatest extent of settlement with both hamlets and a smaller number of large settlement clusters, which are found across a range of elevations and physiographic settings. Several of these settlement clusters were centered on great houses, which are distinctive, multi-storied buildings associated with the expansion of religious and political ideologies from Chaco Canyon. Chaco Canyon (Fig 1) was the prominent Pueblo ritual, political, and economic center in the eleventh and twelfth centuries. Over the course of the 13^th^ century drought, population relocation resulted in the occupation of new areas in the uplands of the region that became viable for cultivation as a result of higher temperatures and drier conditions. Thus in Zuni, climate change opened new places for farming such that per capita arable land was not reduced and may have been enhanced [30, 33]. Through the 13th century, people increasingly clustered in a smaller number of locations, surrounded by largely unoccupied areas. The distances between clusters were relatively small, however, and people remained closely interlinked, regularly exchanging everyday materials like cooking pots [34].

**Fig 2:**
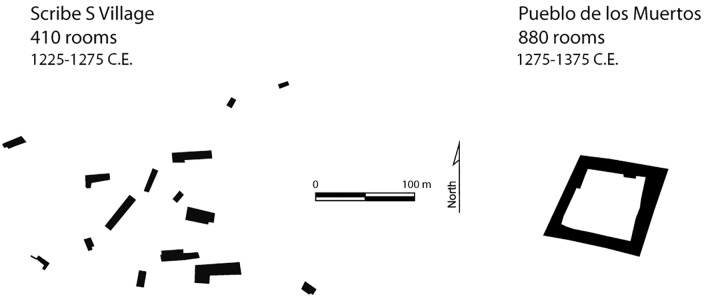
Late 13^th^ Century Shift in Zuni Settlement Organization. Organization shifts from dispersed room blocks (Scribe S) to planned villages (Pueblo de los Muertos).

A few of the largest communities included as many as 500 rooms distributed among closely spaced residential structures, typically ranging from about 2 to 20 rooms. Many of these clusters contained features that harkened back to the earlier Chaco-style great houses, but these late post-Chaco structures included many local innovations in form [35]. The last quarter of the 13th century represented a qualitative shift in settlement organization [25, 34]. Within a single generation, between about 1260 and 1300 C.E., the entire population of the Zuni region reorganized. People went from living in widely distributed small residential structures clustered into communities to massive, planned, nucleated towns containing from about 200 to as many as 1500 rooms within single structures (Fig 2). Room blocks in these large, planned villages were often built around one or more open plazas, and represented a dramatically different scale and form of community. Thus, in the Zuni region, there was continuity of settlement but a transformation in its organization [36]. By 1300 C.E. people lived in a qualitatively different settlement and social system. There is no direct evidence of violence or indirect indications of conflict (e.g., towers and defensive walls) preceding this marked reorganization.

### Mesa Verde

The Mesa Verde region was occupied by Pueblo farmers from the last millennium B.C.E. to the end of the thirteenth century. As with the Zuni case, there were several significant but non-critical transformations in settlement prior to the complete Pueblo depopulation of the region by the late 1200s, which is considered a critical transformation (or regime change) from one state to an alternative. During the mid-to-late 1000s, population increased, settlement distribution expanded, and many communities became focused on Chaco-style great houses. Although population declined somewhat during the severe mid-1100s drought, many who remained in the region relocated to more defensible canyon-oriented settings that were closer to water sources and lived in increasingly aggregated settlements (up to 400–600 rooms) [28, 37, 38]. Population concentrated in the core of the Mesa Verde region, perhaps in part motivated by the fact that it was better watered than many other areas of the northern Southwest. Population growth, concentration, and climate change reduced per capita arable land in the Mesa Verde core as people moved into areas that were already densely occupied. With this increase in population and settlement density, particularly by the mid-1200s, there was increasing diversity in village organization including the use of different religious buildings and village configurations, suggesting experimentation with new social and religious structures. These developments also suggest there were differing ideas about the role of Chaco and its ideologies. The mid-to-late 1200s was a turbulent period with a marked increase in defensive structures, including enclosing walls and especially towers [28, 37], and direct evidence of violence at a number of villages (e.g., [39, 40]). By the end of the thirteenth century all the ancestral Pueblo residents, numbering perhaps as many as 20,000 people in the core area [37], and likely well over 25,000 people in the region [41], emigrated from Mesa Verde. These emigrants and their descendants never returned to live year-round in the Mesa Verde region even though it would have been environmentally possible to farm there.

In sum, both of our cases are examples of marked social transformation, but one entailed continuity in settlement with local organizational changes while the other involved a dramatic regime shift. In Zuni, people remained in place while in Mesa Verde, despite millennia of occupation, an entire population of tens of thousands of people emigrated. Environmental changes occurred in both regions and, yet, although continued settlement in Mesa Verde was possible for at least a part of the population [7, 42], in the end everyone moved out. The explanation for this cannot be purely environmental.

### Southwestern Socio-Ecological System

**Fig 3:**
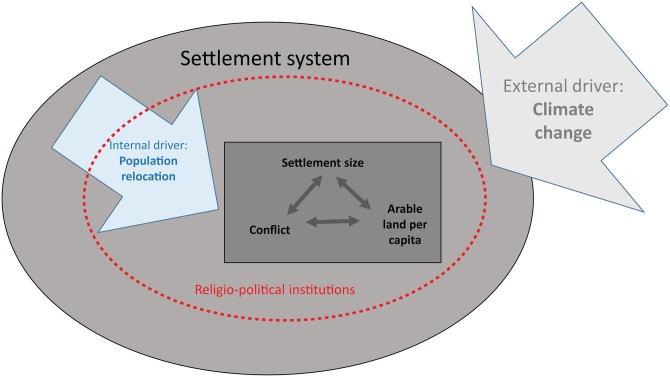
A Conceptual Diagram of a Discrete Socio-ecological System (Mesa Verde or Zuni) in the Late Pre-Hispanic Southwestern US. The diagram shows the system boundary, drivers, and variables.

Fig 3 illustrates a general model of the dynamic socio-ecological system that characterizes our case studies. Table 1 provides information on the external and internal drivers and responses of key internal variables to these drivers. The external driver is climate change. In response to climate change people relocated within the Zuni and Mesa Verde regions. Internal, mutually interacting variables that were affected by both the external driver of climate change and the internal driver of population relocation include the per capita quantity of arable land, settlement size, conflict, and local religio-political institutions and the power and leadership they entailed. Certain settlements grew in size partly due to the availability of arable land but also for protection against conflict and to enhance people’s ability to compete for increasingly scarce patches of agriculturally productive land or permanent sources of water. The relocation of people on both landscapes led to experimentation with new forms of community and leadership. Religio-political leaders created new institutions that are manifest in changes to public architecture in both regions [30, 37, 43]. Some settlements likely grew because these leaders were actively recruiting followers, thereby enhancing their prominence. Leaders and members of these institutions have the potential to mitigate negative factors or to contribute to factionalism, conflict, and ultimately system collapse.

**Table 1:** System Drivers and Variable States in the Case Studies.

External Driver	Mesa Verde	Zuni
Climate change	Severe	Moderate
**Internal Driver**		
Population relocation	Significant	Significant
**Effect of drivers on:**		
Arable land/capita	Reduced	Stable
Settlement size	Variable	Variable
Conflict	Increased (high)	Stable (low)
Leadership/institutions	Increased diversity	Innovation and increased diversity

### Hypotheses

**Fig 4:**
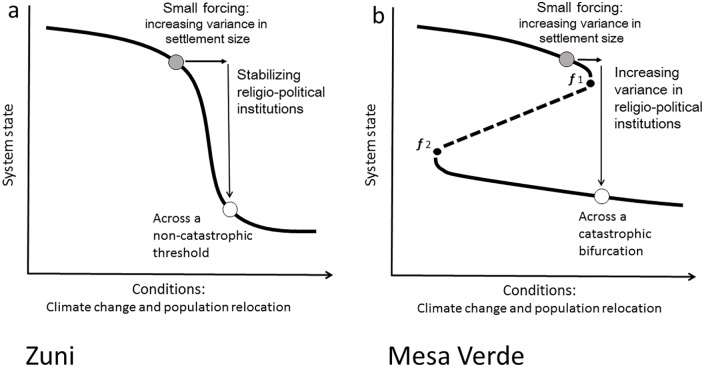
Non-critical and Critical Transformations in the US Southwest. In 4b: *f*1 and *f*2 are fold bifurcation points where a small forcing can cause a sudden qualitative change in a system.

The overarching hypothesis driving this study was that early warning signals are detectable prior to social transformation. Further, we hypothesized that there would be differences between critical and non-critical cases of social transformation in early warning signals and in potential responses to those warnings. Fig 4 summarizes our hypotheses by case study. We began the project with the simple hypothesis that the non-critical transformation, Zuni, would show less variance in settlement size than the critical transformation, Mesa Verde. We then examined social factors that we expected to have played a role in the criticality of the social transformation. Thus our second hypothesis pertained to local religio-political institutions.

Recent literature on early warning signals raises the question as to whether signals can be detected early enough to act upon and prevent regime shift [1, 9, 10, 44, 45]. Although we cannot identify “detection” in the archaeological record, and thus cannot say whether actions that coincide with the early warning signals identified in this analysis are “responses,” we can compare the specific contexts and actions taken with the degree to which a transformation was critical or not.

Some ecological research on SES has emphasized the importance of human management in maintaining system resilience (e.g., [8, 46]). Walker and co-authors [8] note that the “capacity and intent” of human managers strongly influence SES resilience. In the pre-Hispanic US Southwest, religio-political institutions and their leaders would have been a primary locus of community-scale management of social transformation, or a source of social factionalism and cleavage. Factionalism would emerge if a social system became increasingly unstable and existing public social institutions and belief systems were challenged. Past practices and institutions may be revived, existing ones kept but less popular, and new ones developed. Therefore, increasing diversity in religio-political institutions is a potential indicator of factionalism and thus instability in social institutions as people question the status quo and pursue alternatives. Resilience, however, could be enhanced if religio-political institutions were used to dampen social instability. Thus we hypothesised that religio-political institutions were more stable in the Zuni region, than in Mesa Verde, and consequently able to manage social transformation in a way that was not possible in Mesa Verde. Size of public architecture, discussed further in the methods section below, was our proxy for religio-political institutional scale. An increase in variation in public architecture size would indicate increasing diversity in institutional scale and possibly instability in religio-political institutions, while no change or a decrease in CoV would signal stability. We expected that the CoV in Mesa Verde communal structure sizes would be greater than that for similar structures in the Zuni region.

Our third hypothesis also addressed the role of religio-political institutions in social transformation though an assessment of control over ritual practice. Ritual knowledge confers power in Pueblo society [47–49] and secrecy regarding ritual knowledge is a means of controlling and consolidating power [48, 50, 51]. We thus assessed the degree to which ritual leaders sought to control knowledge and thus power by analyzing the types of public architecture found at Zuni and Mesa Verde in terms of public accessibility. Greater conflict, indicative of internal divisions, in the Mesa Verde region led us to expect that it would exhibit more restricted, less inclusive public architecture than Zuni.

## Materials and Methods

### Settlement Size

We assessed settlement size using the number of rooms in habitation structures from several large (> 5 square km) blocks of full-coverage archaeological survey in both the Zuni and Mesa Verde regions representing more than 1,000 habitation structures within each region. The individual sites within each survey were further assigned to specific temporal intervals (“binned”) based on absolute dates and/or the frequencies of temporally sensitive artifacts or features [52, 53]. As site definitions varied across our study areas based on in-field analysts’ decisions to either separate or join individual structures, we developed an additional procedure using ArcGIS to define spatially explicit communities as our units of analysis (see S1 File). Thus, we were able to track change through time in settlement sizes across our case study areas.

### Religio-Political Institutions

**Fig 5:**
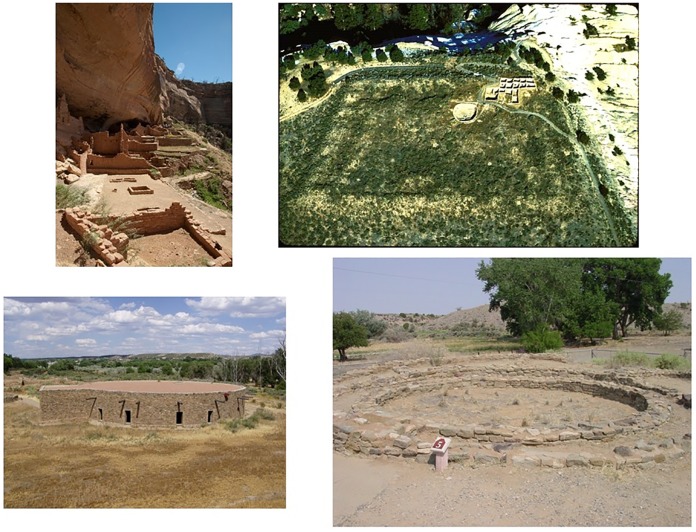
Examples of Southwestern Public Architecture. Clockwise from upper left: Long House Plaza, Mesa Verde [Courtesy of Mesa Verde National Park; photo by Robert D. Jensen]; Atsinna plaza, Zuni [Keith Kintigh]; Hubbard tri-wall, Aztec Ruin National Monument [Donna Glowacki]; reconstructed great kiva, Aztec Ruin National Monument [Matthew Peeples].

In the pre-Hispanic US Southwest public architecture is a primary source of information on social institutions above the level of the household [34, 54, 55]. Most such architecture at least in part is devoted to the preparation for and/or performance of communal rituals. For this project we first relied on the size of public architecture as an indicator of the scale of the organization of social institutions. Different sized public architecture is assumed to indicate different types of ritual organizations in the US Southwest [55, 56]. These buildings include great houses, great kivas, unroofed great kivas, plazas, and in Mesa Verde, multi-walled structures (Fig 5). These features could be assigned temporal intervals of potential use based on material culture and absolute dates. Our public architecture data come from the broader Zuni and Mesa Verde regions, including areas outside of the full-coverage survey blocks (see Tables B and C in S1 File).

The available data allowed us to investigate the CoV of the sizes of great kivas, unroofed great kivas and plazas in the Zuni and Mesa Verde areas to determine whether the social scale of ritual communities increased in variance prior to the transformations in each case. We divided the analysis between communal ritual buildings (great kivas and unroofed great kivas) on the one hand, and communal spaces (plazas) on the other.

Our second source of information on religio-political institutions was the form of public architecture. Following previous analyses [28, 37], we grouped public architecture forms by type: *group assembly* (great kivas and plazas) and *restricted use* (great houses, multi-walled structures, D-shaped structures, and oversized pit structures). *Group assembly* structures could host large numbers of people and are inferred to promote inclusivity. *Restricted use* structures were used by smaller numbers of people and suggest some degree of social exclusivity and control of ritual knowledge. Sample sizes for these types of public architecture were not large enough in all periods to undertake a CoV analysis leading us to rely on raw counts by period.

### Climate

Archaeologists in the US Southwest have not considered drought to be the primary driver of socio-cultural change for quite some time. It is clear that demographic and socio-cultural context are critical in understanding the impacts of climate change on the peoples whom we study. Our hypotheses have to do with early warning signals, and not climate per se, but climate is the primary external driver in our two systems. Thus to provide information on the climatic context of the early warning signals we are investigating, we used the high-quality 2,129-year-long El Malpais precipitation reconstruction data from northwestern New Mexico [57] to represent major trends in climate through time across our case studies. This precipitation reconstruction provides annual estimates of rainfall in inches. We converted these data to Z-scores to illustrate deviations above and below the mean and further calculated a 9-year running mean to capture longer-term trends in rainfall. Further information is provided in the S1 File.

## Results

### Settlement Size

**Fig 6:**
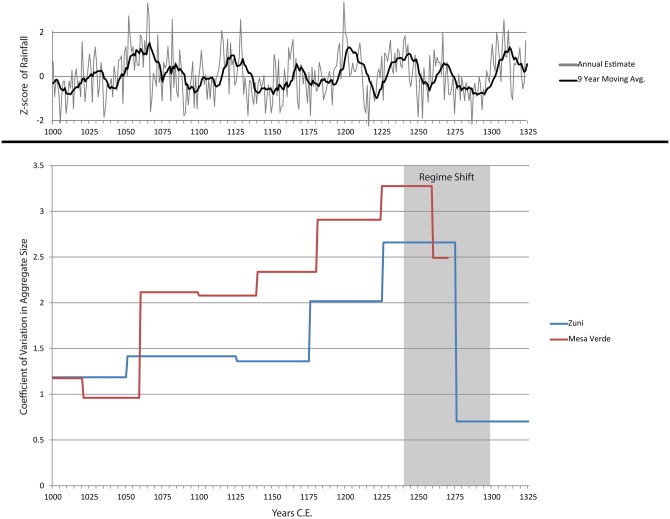
Coefficients of Variation in Settlement Size for Zuni and Mesa Verde Region Sites over Time. Plots of the El Malpais annual precipitation reconstruction and of the 9-year running mean are provided at the top of the figure to illustrate climate trends during this period.

Fig 6 displays the coefficients of variation (CoV) in settlement size for the Zuni and Mesa Verde cases. The El Malpais precipitation reconstruction and dry period calculations are illustrated at the top of the figure. The precipitation data are annual, while the CoV data are calculated by 50-year intervals, hence the stepped pattern in that portion of the figure. The El Malpais data illustrate that the external driver of climate delivered repeated stresses to these regions of the US southwest in the late pre-Hispanic period. Settlement changes, however, are varied and do not always occur during or immediately following dry periods.

Both plots show that the relative amount of variation in aggregate size increased dramatically prior to each transformation; however, the peak CoV for Mesa Verde is greater than that for Zuni. In the Zuni region, the peak in CoV in the early-to-mid 1200s is due to a small number of population aggregates increasing dramatically in size, the very first nucleated villages that predate 1275 C.E., while most villages remained small. In the Mesa Verde region, two periods of increasing variance prior to major settlement transformations are captured: 1) a rapid increase in aggregate size in the late 1000s due to immigration [7, 38], *in situ* population growth, and the establishment of great house communities; and 2) the peak in CoV in the mid-1200s (similar in timing to the Zuni region) due to marked population growth, internal population movement, and increased site density, which included the development of new types of village configuration and increased variation in settlement sizes [28, 37, 55]. After 1250 C.E., the rate of emigration from the Mesa Verde region increased significantly, and other behavioral changes discussed below indicate the mid-1200s was the tipping point for Mesa Verde. Further comparative analyses of variance prior to threshold changes will be necessary before we understand whether there is a relationship between absolute differences in the magnitude of the CoVs and the degree of criticality in the transformation.

### Religio-Political Institutions

**Fig 7:**
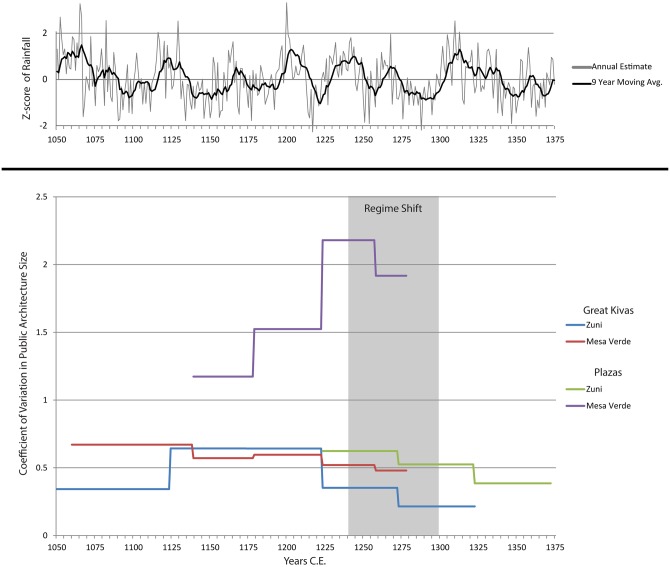
Coefficients of Variation for Zuni and Mesa Verde Great Kiva and Plaza Space over Time. Plots of the El Malpais annual precipitation reconstruction and of the 9-year running mean are provided at the top of the figure to illustrate climate trends during this period.

Fig 7 shows that the coefficient of variation in size of great kivas and unroofed great kivas in the Zuni region was relatively stable over time, although it did increase slightly in the early 1100s to early 1200s C.E. when roofed and unroofed forms co-existed. The CoV for Mesa Verde great kivas is perhaps even more stable than Zuni, though this likely relates to the longevity of occupation of these settlements. Plaza size CoVs, however, differ markedly between the two areas. As Fig 7 indicates, Zuni plaza size variation was minimal, while that of late period Mesa Verde plazas was much higher than any form of Zuni public architecture and increased markedly in the mid-1200s, even as emigration from the region was increasing. Formal plazas developed later in time and are much more diverse in the Mesa Verde area than in Zuni (where villages typically had only one or two central plazas). Mesa Verde plazas are more often associated with specific religious buildings or room blocks, suggesting that their size is a reasonable index of variation in internal religio-political organization size. As with the settlement size data, variation in the size of communal ritual structures does not track broad patterns of variation in climate.

With respect to institutional accessibility and control of ritual knowledge, both the Zuni (Table 2) and Mesa Verde (Table 3) regions exhibit a trajectory toward greater inclusivity in the size and visual accessibility (roofed great kivas, to unroofed great kivas, to large plazas) of group assembly public architecture over time [34, 37]. In addition in Zuni, after the establishment of large planned settlements, restricted-use public architecture declined dramatically [34] likely indicating a reduction in the emphasis on exclusivity in ritual practice and participation. In the Mesa Verde core area, however, restricted-use structures continued to be used until abandonment. Rather than a reduction, there was a reorientation and revitalization of restricted-use architecture. Great houses continued to be used, but in different ways, and multi-walled structures were introduced and elaborated upon [37, 58]. In addition, the distributions of particular forms of multi-walled structures (circular versus D-shaped) were mutually exclusive, possibly representing the development of distinct religious factions that contributed to larger rifts among the Mesa Verde centers [37].

**Table 2:** Frequency of Zuni Communal Structure Types.

Period	Restricted-Use	Group Assembly	Total	% Restricted Use
1050–1125 C.E.	35	22	57	61
1125–1175 C.E.	20	15	35	57
1175–1225 C.E.	25	15	40	63
1225–1275 C.E.	23	26^a^	49	47
1275–1325 C.E.	0	27	27	0

Data are provided by period using peak occupation phase of sites.

^a^Possibly more early plazas but site dating unclear.

**Table 3:** Frequency of Central Mesa Verde Communal Structure Types.

Period	Restricted-Use	Group Assembly	Total	% Restricted Use
1060–1100 C.E.	5	3	8	63
1100–1140 C.E.	7	5	12	58
1140–1180 C.E.	3	4	7	43
1180–1225 C.E.	5	5	10	50
1225–1260 C.E.	21	22	43	49
1260–1280 C.E.	8	10	18	44

Data are provided by phase using peak occupation phase of sites.

## Discussion

Our analyses support our overarching position that early warning signals can be detected prior to social transformation. In both the critical and non-critical transformation, variance in settlement size markedly increased prior to transformation. Our three hypotheses concerning differences in early warning signals between a critical and a non-critical case of social transformation were also upheld. The CoV of settlement size in Mesa Verde was substantially greater than that for Zuni; the CoV for plaza size was also substantially greater for Mesa Verde. While Zuni people dispensed with restricted public architecture and increasingly built inclusive ritual spaces, Mesa Verde ritual organizations maintained some degree of emphasis on exclusive ritual space and presumably ritual action. Moreover, Glowacki [37] has documented the differential distribution of different types of multi-walled structures, indicating a growth in factionalism in religio-political institutions in 13^th^ century Mesa Verde. Our study implicates such factionalism and an emphasis on hierarchical organization in the catastrophic regime change experienced by Mesa Verde peoples. Overall, the critical transformation was characterized by social fragmentation and inter-personal violence and the non-critical one by social stability and inclusivity.

A recent paper by Bocinsky and colleagues [59] documents that marked demographic and cultural (ritual, architectural, material cultural) changes are associated with transitions across cultural phases in the US Southwest (e.g., Pueblo III to Pueblo IV) that were defined by archaeologists almost a century ago. Our study shows that these changes are not just the hallmarks of cultural transition but that demographic and cultural variance can in fact be early warning signals that social transformation is about to take place.

Long-term history clearly plays a role in understanding the context in which critical transformations take place. As Fig 6 indicates, the Mesa Verde area experienced a marked increase in variance in community size in the mid-1000s C.E. at a time of dramatic population influx, and variance remained high even when immigration declined. Thus, coming into the droughts of the mid 1100s and 1200s, this region was already less stable than Zuni. In social systems, then, it is clear that historical processes affect how societies respond to early warnings.

This paper captures the primary trends in settlement and religio-political institution change that pertain to early warning signals prior to the social transformations at Zuni and Mesa Verde. In the course of analyzing these changes we have come to understand that finer-grained processes were at work as well, such as differences in the relevance of Chaco to our two cases, which we will pursue in further research. Chaco may have been an important external driver for Mesa Verde but was not for Zuni, amplifying the differences in religio-political institutions that may be implicated in differences in social stability between the two regions.

## Conclusions

A wealth of well-dated archaeological settlement and architectural data from the US Southwest has allowed us to investigate whether the concept of early warning signals for transformations is applicable to social systems. The results of our analyses suggest that this approach is useful in understanding social transformation. Moreover, given that the archaeological record contains information on completed cycles of critical and non-critical threshold change, it provides important data for new comparative analyses of the contexts of and apparent responses to these signals that can illuminate the study of early warning signals in contemporary social systems and ways in which to manage threshold change.

In both archaeological cases, directional drivers of increasing stress (climate change and population relocation) were followed by greater variation in the size of settlements people chose to live in, with some settlements increasing dramatically in size. That variance presaged transformation in both cases. There were large differences, however, in the absolute scale of variation between the cases. These differences raise the issue of whether there is an absolute value of variance above which a transformation becomes critical. Biggs and co-authors [10], for example, suggest that research should ultimately be able to identify such critical indicator levels. Although Brock and co-authors [17] have cautioned that no single threshold level will apply to all cases of a system, they encourage extensive comparative analyses so that the system of interest may be appropriately divided into subsets relevant to driving variables. Our research begins that kind of comparative analysis for late pre-Hispanic SES in the US Southwest.

Unlike the responses of ecological systems to drivers of system change, social systems contain institutions that can both be used to manage responses (stabilize), and to introduce instability. By taking a socio-ecological perspective and including analyses of different aspects of social institutions, we have identified marked differences in how each society handled increasing instability. Communal social institutions in the non-critical transformation (Zuni) appear to have managed regime change by promoting inclusive communal action and reducing exclusionary actions and overt hierarchy. In contrast, in the critical transformation (Mesa Verde), hierarchy, instability and conflict in social institutions appear to have added to the stress already experienced with drought and population packing.

This emphasis on the capacity of social institutions to mitigate transformation brings us to our final point. The current early warning signals literature is heavily focused on ecological systems and humans are generally perceived either as external sources of disturbance or external forces for management. Some ecologists express concern that society may not have the traditions, policies, and institutions necessary to take appropriate action in time to prevent critical ecological transformations [10, 17]. Including the societal component of socio-ecological systems will be critical in deducing what actions in fact are considered appropriate and what social and political processes and institutions are available to take those actions. The people who formed the communal social institutions that we analyzed for this paper were deeply embedded in the Zuni and Mesa Verde socio-ecological systems and thus knowledgeable of the effects of long-term drought on their crops, where people were living and why, and the degree to which their social relations were fractured or coherent. A strong integration of ecological and social science research is critical for effectively using the early warning signals research that ecologists have pioneered.

## Supporting Information

S1 File Supporting Information. (DOCX)

## References

[1] Scheffer M, Carpenter SR, Lentor TM, Bascompte J, Brock W, Dakos V, et al Anticipating critical transitions. Science. 2012;338(344): 344–348. 10.1126/science.1225244 23087241

[2] Turner BL III, Sabloff JA. Classic period collapse of the Central Maya Lowlands: insights about human-environment relationships for sustainability. Proc Natl Acad Sci USA. 2012;109(35): 13908–13914. 10.1073/pnas.1210106109 22912403 PMC3435155

[3] Costanza R, Graumlich L, Steffen W, Crumley C, Dearing J, Hibbard K, et al Sustainability or collapse: what can we learn from integrating the history of humans and the rest of nature? Ambio. 2007;36(7): 522–527. 10.1579/0044-7447(2007)36[522:SOCWCW]2.0.CO;2 18074887

[4] McAnany P, Yoffee N. Questioning collapse: Human resilience, ecological vulnerability, and the aftermath of empire. Cambridge: Cambridge University Press; 2009.

[5] Tainter J. Archaeology of overshoot and collapse. Annu Rev Anthropol. 2006;35: 59–74. 10.1146/annurev.anthro.35.081705.123136

[6] Nelson MC, Ingram SE, Dugmore AJ, Streeter R, Peeples MA, Mcgovern TH, et al Climate challenges, vulnerabilities, and food security. Proc Natl Acad Sci USA. 2016;113(2): 298–303. 10.1073/pnas.1506494113 26712017 PMC4720298

[7] Schwindt DM, Bocinsky RK, Ortman SG, Glowacki DM, Varien MD, Kohler TA. The social consequences of climate change in the central Mesa Verde region. Am Antiq. 2016;81(1): 74–96. 10.7183/0002-7316.81.1.74 PMC7523884 33001060

[8] Walker B, Holling CS, Carpenter SR, Kinzig A. Resilience, adaptability, and transformability in social-ecological systems. Ecol Soc. 2004;9(2): article 5.

[9] Scheffer M, Bascompte J, Brock WA, Brovkin V, Carpenter SR, Dakos V, et al Early-warning signals for critical transitions. Nature. 2009;461(7260): 53–59. 10.1038/nature08227 19727193

[10] Biggs R, Carpenter SR, Brock WA. Turning back from the brink: detecting an impending regime shift in time to avert it. Proc Natl Acad Sci USA. 2009;106 (3): 826–831. 10.1073/pnas.0811729106 19124774 PMC2630060

[11] Van Nes EH, Scheffer M. Slow recovery from perturbations as a generic indicator of a nearby catastrophic shift. Am Nat. 2007;169(6): 738–747. 10.1086/516845 17479460

[12] Kefi S, Dakos V, Scheffer M, Van Nes EH, Rietkerk M. Early warning signals also precede non-catastrophic transitions. Oikos. 2013;122: 641–648. 10.1111/j.1600-0706.2012.20838.x

[13] Wissel C. A universal law of the characteristic return time near thresholds. Oecologia. 1984;65: 101–107. 10.1007/BF00384470 28312117

[14] Dakos V, Scheffer M, van Nes EH, Brovkin V, Petoukhov V, Held H. Slowing down as an early warning signal for abrupt climate change. Proc Natl Acad Sci USA. 2008;105(38): 14308–14312. 10.1073/pnas.0802430105 18787119 PMC2567225

[15] Dakos V, Carpenter SR, Brock WA, Ellison AM, Guttal V, Ives AR, et al Methods for detecting early warnings of critical transitions in time series illustrated using simulated ecological data. PLoS One. 2012;7(7): e41010 10.1371/journal.pone.0041010 22815897 PMC3398887

[16] Brock WA, Carpenter SR. Interacting regime shifts in ecosystems: implication for early warnings. Ecol Monogr. 2010;80(3): 353–367. 10.1890/09-1824.1

[17] Brock WA, Carpenter SR, Scheffer M. Regime shifts, environmental signals, uncertainty, and policy choice In: Norberg J, Cumming GS, editors. Complexity theory for a sustainable future. New York: Columbia University Press; 2008 pp. 180–206.

[18] Dakos V, Kefi S, Rietkerk M, van Nes EH, Scheffer M. Slowing down in spatially patterned ecosystems at the brink of collapse. Am Nat. 2011;177(6): E153–E166. 10.1086/659945 21597246

[19] Ditlevson PD, Johnsen SJ. Tipping points: early warning and wishful thinking. Geophys Res Lett. 2010;37: L19703 10.1029/2010GL044486

[20] Carpenter SR, Brock WA. Rising variance: A leading indicator of ecological transition. Ecol Lett. 2006; 9: 311–318. 10.1111/j.1461-0248.2005.00877.x 16958897

[21] Lipe WD, Varien MD, Wilshusen RH. Colorado prehistory: A context for the southern Colorado River basin. Denver: Colorado Council of Professional Archaeologists and State Historical Fund, Colorado Historical Society; 1999.

[22] Kintigh KW. Settlement, subsistence, and society in late Zuni prehistory. Tucson: University of Arizona Press; 1985.

[23] Nelson MC, Kintigh KW, Abbott DR, Anderies JM. The cross-scale interplay between social and biophysical context and the vulnerability of irrigation-dependant societies: archaeology’s long-term perspective. Ecol Soc. 2010:15(3): article 31.

[24] Varien MD. Sedentism and mobility in a social landscape: Mesa Verde and beyond. Tucson: University of Arizona Press; 1999.

[25] Kintigh KW, Glowacki DM, Huntley DL. Long-term settlement history and the emergence of towns in the Zuni area. Am Antiq. 2004;69(3): 432–456. 10.2307/4128401

[26] Van West CR, Dean JS. Environmental characteristics of the A.D. 900–1300 period in the Central Mesa Verde region. Kiva. 2000; 66(1): 19–44.

[27] Wright A. The climate of the depopulation of the Northern Southwest In: Kohler T, Varien M, Wright A, editors. Leaving Mesa Verde: Peril and change in the thirteenth-century southwest. Tucson: University of Arizona Press; 2010 pp. 75–101.

[28] Glowacki DM, Ortman SG. Characterizing community center (village) formation in the VEP study area, A.D. 600–1280 In: Kohler TA, Varien MD, editors. Emergence and collapse of early villages: Models of central Mesa Verde archaeology. Berkeley: University of California Press; 2012 pp. 219–246.

[29] Varien MD, Lipe WD, Adler MA, Thompson IM, Bradley BA. Southwestern Colorado and southeastern Utah settlement patterns: A.D. 1100 to 1300 In: Adler MA, editor. The prehistoric Pueblo world, A.D. 1150–1350. Tucson: University of Arizona Press; 1996 pp. 86–113.

[30] Schachner G. Population circulation and the transformation of ancient Cibola communities. Tucson: University of Arizona Press; 2012.

[31] Peeples MA. Population history of the Zuni region across the protohistoric transition: migration, gene flow, and social transformation In Villalpando E, McGuire RH, editors. Building transnational archaeologies. Tucson: Arizona State Museum Archaeological Series 209; 2014 pp. 93–109.

[32] Ferguson TJ, Hart ER. A Zuni atlas. Norman: University of Oklahoma Press; 1985.

[33] Van West CR, Grissino-Mayer HD. Dendroclimatic reconstruction In: Huber EK, Van West CR editors. Fence Lake Project: Archaeological data recovery in the New Mexico transportation corridor and first five-year permit area, Fence Lake Coal Mine Project, Catron County, New Mexico, Volume 3: Environmental studies. Tucson: Statistical Research, Inc; 2005 pp. 33.1–33.130.

[34] Peeples MA. Identity and social transformation across the pre-Hispanic Cibola world: A.D. 1150–1325. PhD Dissertation, Arizona State University. 2011.

[35] Kintigh KW, Howell TL, Duff AI. 1996 Post-Chacoan social integration at the Hinkson site, New Mexico. Kiva. 1996;61(3): 257–274.

[36] Watson PJ, LeBlanc SA, Redman CL. Aspects of Zuni prehistory: preliminary report on excavations and survey in the El Morro Valley of New Mexico. J Field Archaeol. 1980;7: 201–218. 10.2307/529759

[37] Glowacki DM. Living and leaving: A social history of regional depopulation in thirteenth-century Mesa Verde. Tucson: University of Arizona Press; 2015.

[38] Varien MD, Ortman SG, Kohler TA, Glowacki DM, Johnson CD. Historical ecology in the Mesa Verde region: results from the Village Ecodynamics Project. Am Antiq. 2007;72(2): 273–299. 10.2307/40035814

[39] Kuckelman KA. The depopulation of Sand Canyon Pueblo, a large Ancestral Pueblo village in southwestern Colorado. Am Antiq. 2010;75(3): 497–526. 10.7183/0002-7316.75.3.497

[40] Kuckelman KA. Catalysts of the thirteenth-century depopulation of Sand Canyon Pueblo and the central Mesa Verde region In: Kohler T, Varien M, Wright A, editors. Leaving Mesa Verde: Peril and change in the thirteenth-century southwest. Tucson: University of Arizona Press; 2010 pp. 180–199.

[41] Kohler TA, Varien MD, Wright AM. Leaving Mesa Verde: Peril and change in the thirteenth-century Southwest. Tucson: University of Arizona Press; 2010.

[42] Van West CR. Modeling prehistoric agricultural productivity in southwestern Colorado: A GIS approach. Pullman: Department of Anthropology Reports of Investigations 67; 1994.

[43] Duff AI, Schachner G. Becoming central In: Sullivan API, Bayman JM, editors. Hinterlands and regional dynamics in the ancient Southwest. Tucson: University of Arizona Press; 2007 pp. 185–200.

[44] Carpenter SR, Cole JJ, Pace ML, Batt R, Brock WA, Cline T, et al Early warnings of regime shifts: a whole-ecosystem experiment. Science. 2011;332(1079): 1079–1082. 10.1126/science.1203672 21527677

[45] Scheffer M, Westley F, Brock W. Slow responses of societies to new problems: causes and costs. Ecosystems. 2003;6: 493–502. 10.1007/PL00021504

[46] Folke C, Carpenter S, Elmqvist T, Gunderson L, Holling CS, Walker B. Resilience and sustainable development: building adaptive capacity in a world of transformations. Ambio. 2002;31(5): 437–440. 10.1579/0044-7447-31.5.437 12374053

[47] Ortiz A. The Tewa world; space, time, and becoming in a Pueblo society. Chicago: University of Chicago Press; 1969.

[48] Brandt EA. Egalitarianism, hierarchy, and centralization in the Pueblos In: Wills WH, Leonard RD, editors. The ancient southwestern community. Albuquerque: University of New Mexico Press; 1994 pp.9–23.

[49] Ware JA. A Pueblo social history: Kinship, sodality, and community in the northern Southwest. Santa Fe: School of Advanced Research Press; 2014.

[50] Brandt EA. The role of secrecy in a Pueblo society In: Blackburn TC, editor. Flowers of the wind: Papers on ritual, myth, and symbolism in California and the southwest. Socorro, NM: Ballena Press; 1977 pp. 11–28.

[51] Brandt EA. On secrecy and control of knowledge In: Tefft S, editor. Secrecy: A cross-cultural perspective. New York: Human Sciences Press; 1980 pp. 123–146.

[52] Ortman SG, Varien MD, Gripp TL. Empirical Bayesian methods for archaeological survey data: an application from the Mesa Verde region. Am Antiq. 2007;72(2): 241–272. 10.2307/40035813

[53] Peeples MA, Schachner G. Refining correspondence analysis-based ceramic seriation of regional data sets. J Archaeol Sci. 2012;39: 2818–2827. 10.1016/j.jas.2012.04.040

[54] Lipe WD. Social power in the central Mesa Verde region, AD 1150–1290 In: Varien MD, Wilshusen RH, editors. Seeking the center place: Archaeology and ancient communities in the Mesa Verde region. Salt Lake City: University of Utah Press; 2002 pp. 203–232.

[55] Adler MA, Wilshusen RH. Large-scale integrative facilities in tribal societies: cross-cultural and southwestern U.S. examples. World Archaeol. 1990;22(2): 133–146. 10.1080/00438243.1990.9980136

[56] Lekson SH. Great House form In: Lekson SH, editor. The architecture of Chaco Canyon, New Mexico. Salt Lake City: University of Utah Press; 2007 pp. 7–44.

[57] Grissino-Mayer HD. A 2129-year reconstruction of precipitation for northwestern New Mexico, U.S.A In: Dean JS, Meko DM, Swetnam TW, editors. Tree rings, environment and humanity. Radiocarbon 1996, Department of Geosciences. Tucson: University of Arizona; 1996 pp. 191–204.

[58] Lekson SH, Cameron CM. The abandonment of Chaco Canyon, the Mesa Verde migrations, and the reorganization of the Pueblo world. J Anthropol Archaeol. 1995;14: 184–202. 10.1006/jaar.1995.1010

[59] Bocinsky RK, Rush J, Kintigh KW, Kohler TA. Exploration and exploitation in the macrohistory of the pre-Hispanic Pueblo southwest. Sci Adv. 2016; 2: e1501532 10.1126/sciadv.1501532 27051879 PMC4820384

