# Supplementary material for: Early Warning Signals of Social Transformation: A Case Study from the US Southwest
Source: PLoS One. 2016 Oct 5;11(10):e0163685. doi: 10.1371/journal.pone.0163685 (PMC5051805; doi:10.1371/journal.pone.0163685)
Supplement: S1 File — (DOCX) [file pone.0163685.s001.docx]

**Supporting Information**

**Text A: Materials and Methods**

**Defining Population Aggregates**. To evaluate the relationship between diversity in settlement size and transformations in regional settlement distribution through time, it was first necessary to define the appropriate scale for our unit of analysis (i.e., the local settlement, or community). In the northern Southwest the distribution of room blocks can vary dramatically from place to place. In some locations, individual structures are relatively isolated, whereas in others dense clusters of structures are found in close association and likely represent communities of frequently interacting households. As we are interested in tracking changes in the size of local communities, it is these clusters, rather than individual room blocks, that are of interest. Regional settlement data provided the information on which our analysis was based.

To define settlement clusters we drew on recent work by Peterson and Drennan [1] that focused on identifying communities from large-scale, full-coverage, regional scale survey data. Such regional settlement data provide information on the degree and nature of "clustering" of settlements across a landscape. Although communities are often not strictly defined in terms of spatial distances among people, the scale at which settlement is clustered in a particular setting provides information on the likely intensity of local, face-to-face interactions (i.e., our assumption is that greater spatial proximity likely indicates a higher frequency of interaction). The approach advocated by Peterson and Drennan [1, 2] uses iterative analyses and visual inspection of contour maps of settlement locations and size (at various levels of smoothing) across space to identify the scale or scales marked by the greatest degree of local clustering for a given setting in order to define the appropriate units (local communities) for comparative analysis. In this study, we built on the methods developed by Peterson and Drennan to define likely local communities which allowed us to consistently examine variation in population aggregate size through time. Because our study areas are marked by deep canyons, tall mesas, and other rugged terrain that likely would have constrained foot travel and interaction among the residents of various locations, we have adapted this approach to assess settlement clustering based on the costs associated with travel based on topography [3, 4]. This approach is similar, in many ways, to recent approaches to defining settlement clusters presented by Mills and colleagues [5] but is focused on a smaller spatial scale.

Briefly, our approach consisted of creating concentric buffers around individual room blocks based on the cost of traversing the terrain in 100 cost-equivalent meter intervals up to 1 kilometer (1 km being Peterson and Drennan's [1] estimated likely maximal scale of face-to-face community interaction). Cost equivalent distance, in this case, is defined based on the effort required to traverse variable terrain in relation to the estimated effort of traversing a given distance on a flat plain. This analysis used a 30-m resolution digital elevation model and previously compiled information on the average height and weight of a person living in the Puebloan Southwest during the period considered here. The specific cost model is described in detail elsewhere [3]. We do not suggest that these cost equivalent distances necessarily reconstruct exact paths walked by individuals in the past. We do, however, suggest that they provide a better indication of likely corridors of movement than linear distances alone.

Once cost equivalent buffers around all sites were created (in 100 meter equivalent intervals), we could then assess the degree to which sites cluster. Where two or more room blocks share overlapping buffers at a given cost distance, they were considered “clustered” at that level. We could then count the total number of rooms (and by extension population) represented by clusters across a range of distances to track population density at various spatial scales. For example, in Fig A it is clear that the Lower Zuni area is marked by a greater degree of spatial clustering at shorter distances than the Pescado Basin area within the Zuni region where room blocks are more dispersed. We can further quantify this difference by comparing the mean size (in terms of room count) of clusters or aggregates at a given cost distance. As the line plot shows, the average size of aggregates increases steadily in both areas as the distance considered increases. Importantly, however, the Lower Zuni area in this comparison is marked by a considerably greater degree of clustering across all distances considered (see also [6]).

Using the approach described above we produced maps for a number of large, contiguous, non-linear full-coverage survey areas in both the Mesa Verde Core (VEP I study area; [7]) and Zuni [8-11] regions for each of the temporal intervals for which we have data (Figs B-Q). Each of these maps for both the Mesa Verde and Zuni region are provided in this supplement. Drawing on heuristics provided by Peterson and Drennan [1], we then used the distribution of room blocks across buffers of various sizes to determine the distances marked by the greatest degree of concentration and clustering. In order to do this, we examined maps for all time periods visually as well as histograms of room counts across all buffer distances. As Peterson and Drennan [1] note, the selection of buffer distances is a subjective process, but one that is guided by ethnographic expectations concerning the likely size of face-to-face communities and easily allows the rejection of distances not marked by coherent clusters. Following their published suggestions, we determined the most appropriate distances for defining our local clusters in both focal areas such that the clusters produced were of a similar scale across multiple survey areas within each region, while also avoiding distances that produced long, linear clusters of room blocks. In general similar cost-distance thresholds of 200 meters (Zuni) and 300 meters (Mesa Verde) appear to produce consistent results across all time periods considered. Importantly, these distances are comparable to those identified by Peterson and Drennan [1:11] across a range of societies and survey areas where the buffers selected were typically a few hundred meters in extent.

We examined variation in cluster sizes through time by calculating the coefficient of variation in cluster size using total room count within each buffer for each time period in both study regions. If the early signals model holds for changes in settlement size and distribution through time, we would expect an increase in the coefficient of variation in cluster size prior to periods of transformation in each case. Table A contains summary statistics for settlement size by period for each case study.

Given differences in the nature of archaeological survey data between the Zuni and Mesa Verde regions, it is also important to assess the degree to which survey boundaries themselves may influence differences in the coefficients of variation in settlement size as defined above. In both study areas, survey blocks typically consist of large, full-coverage surveys in continuous blocks ranging from several hundred to more than 10,000 acres. As Fig R illustrates, survey blocks are considerably larger than the relatively short cost-equivalent distances used to define clusters. Notably, however, Fig R also illustrates one potential difference between survey blocks in the two study areas. The survey boundaries for Mesa Verde most often consist of large blocks including both canyons and mesa tops whereas many of the Zuni area survey areas are restricted primarily to canyon bottoms and mesa slopes with less coverage of mesa top settings. Past regional survey research in the Zuni region has demonstrated that habitation sites are most often located in such mesa slope settings [8, 9] but it is also likely that additional survey on mesa tops would reveal more structures. If additional mesa top sites (which are typically small) were identified, this could potentially increase the coefficient of variation in settlement size. In order to assess the potential impact of undercounting sites in the Zuni sample, we conducted a series of experimental calculations using different assumptions about the range of missing settlements to determine the possible magnitude of changes in variation we might reasonably expect due to missing sites. These calculations suggest that the overall results are robust, even when we assume an unreasonably high number of missing settlements. For example, in the Zuni area survey block shown in Fig R, there are 49 settlement clusters. If we were to assume that additional survey of mesa tops and other adjacent areas revealed an additional 25 structures of the modal size for that period of 5 rooms (a more than 50% increase in the number of clusters) the coefficient of variation for that survey block would change from 1.46 to 1.61. This increase in the number of settlements, which is considerably higher than what we would expect based on existing survey data of mesa top settings, still only results in a 10.2% increase in the coefficient of variation. The differences in coefficients of variation between the Zuni and Mesa Verde regions are more than twice that for most periods (for example, > 23% during the period of regime change). We found similar patterns when we conducted similar exercises in other time periods and survey areas. Overall, this suggests that, although differences in survey blocks between the two study areas may influence our calculations somewhat, the degree of variation between the two study areas is still greater than the differences we might expect under a reasonable range of assumptions of additional missing settlements.

**Public Architectural Data.** The identification of specific classes of communal ritual features is based on information recorded in the field for each site; sizes were determined from detailed scale maps of public structures. Tables B and C present the public architectural data size estimates used here. These data were previously compiled by Peeples [12] for the Zuni area and include all great kivas with estimates of diameter that overlap with the period in question (1000-1350 C.E.). Area estimates were derived from these linear diameter estimates based on excavation information or the size of the surface depression in cases where excavation data were not available. Plazas areas were measured using scaled maps provided by Kintigh [13] of several large Pueblo IV period villages in the Zuni region. Measurements were made using the Polygon measurement tool in Image J (version 1.47) by drawing the largest possible interior polygon within unroofed open space according to the site plan drawings.

For the Mesa Verde area, data on great kiva and plaza areas are currently only available for 24 Pueblo III period community centers. Therefore, the Zuni and Mesa Verde public architecture data are not completely analogous. Grant Coffey (Crow Canyon Archaeological Center) collected the Mesa Verde data using scaled, vector-based maps in AutoCAD to calculate great kiva and plaza area. Great kivas were measured using linear diameter based on the size of surface depression, and in some cases based on excavation data. In the Mesa Verde region, plazas, defined as extramural spaces bounded by architecture on at least two sides, are quite variable in their formality, location within the village, and proximity to other public architecture or residential architecture.

**Climatic Data.** In order to characterize changes in climatic conditions through time we relied on the high quality 2,129 year precipitation reconstruction of precipitation in northwestern New Mexico developed by Henri Grissino-Mayer [14]. This dendroclimatological reconstruction provides annual estimates of rainfall in inches from 136 B.C. to A.D. 1992. For the purposes of this analyses, we used rainfall estimates for the 500 year period from A.D. 900-1399 to bracket the period considered in both the Zuni and Mesa Verde cases. We then converted the annual rainfall for the selected interval into Z-score values where 0 represents the mean for the entire 500-year interval and values above and below 0 represent deviations above and below the long-term mean in standard deviation units. We then calculated a 9-year running mean from these standardized annual rainfall values in order to capture longer-term trends using methods defined by Ingram [15]. Overall, this procedure allows us to identify and evaluate prolonged periods of below average precipitation of a severity and duration that likely would have negatively impacted agricultural productivity.

**SI References**

1. Peterson CE, Drennan RD. [Communities, settlements, sites, and surveys: regional-scale analysis of prehistoric human interaction](http://www.jstor.org/stable/40035266). Am Antiq. 2005;70(1): 5-30.

2. Drennan, RD, Peterson, CE. Patterned variation in prehistoric chiefdoms. Proc Natl Acad Sci USA. 2006;103(11): 3960-3967*.*

3. Herhahn C, Hill JB. Modeling agricultural production strategies in the northern Rio Grande Valley, New Mexico. Hum Ecol. 1998;26: 469–487.

4. Varien MD. Sedentism and mobility in a social landscape: Mesa Verde and beyond. Tucson: University of Arizona Press; 1999.

5. Mills BJ, Clark JL, Peeples MA, Haas WR, Roberts JM, Hill JB, et al. The transformation of social networks in the late prehispanic U.S. Southwest. Proc Natl Acad Sci USA. 2013;110 (15): 5785-5790.

6. Peeples MA, Schachner G. Refining correspondence analysis-based ceramic seriation of regional data sets. J Archaeol Sci. 2012; 39: 2818-2827.

7. Kohler TA, Varien MD. Emergence and collapse of early villages: Models of central Mesa Verde archaeology*.* Berkeley: University of California Press; 2012.

8. Kintigh KW, Glowacki DM, Huntley DL. Long-term settlement history and the emergence of towns in the Zuni area. Am Antiq. 2004;69(3): 432-456.

9. Kintigh KW. Late prehistoric and protohistoric settlement systems in the Zuni area. In: Gregory DA, Wilcox DR editors. Zuni origins: Toward a new synthesis of Southwestern archaeology. Tucson: University of Arizona Press; 2007. pp. 361-376.

10. Schachner G. Population circulation and the transformation of ancient Zuni communities Tucson: University of Arizona Press; 2012.

11. Watson PJ, LeBlanc SA, Redman CL. Aspects of Zuni prehistory: Preliminary report on excavations and survey in the El Morro Valley of New Mexico. J Field Archaeol. 1980:7: 201-218.

12. Peeples MA. Identity and social transformation across the pre-Hispanic Cibola world: A.D. 1150-1325. PhD Dissertation, Arizona State University. 2011.

13. Kintigh KW. Settlement, subsistence, and society in late Zuni prehistory. Tucson: University of Arizona Press; 1985.

14. Grissino-Mayer, HD. A 2129 Year annual reconstruction of precipitation for northwestern New Mexico, USA. In: Dean, JS, Meko DM, Swetnam TW, editors. Tree rings, environment, and humanity. Radiocarbon 1996, Department of Geosciences. Tucson: University of Arizona; 1996. pp. 191-204.

15. Ingram, SE. Human vulnerability to climatic dry periods in the prehistoric U.S. Southwest. PhD Dissertation, Arizona State University. 2010.

**S1 Figures**


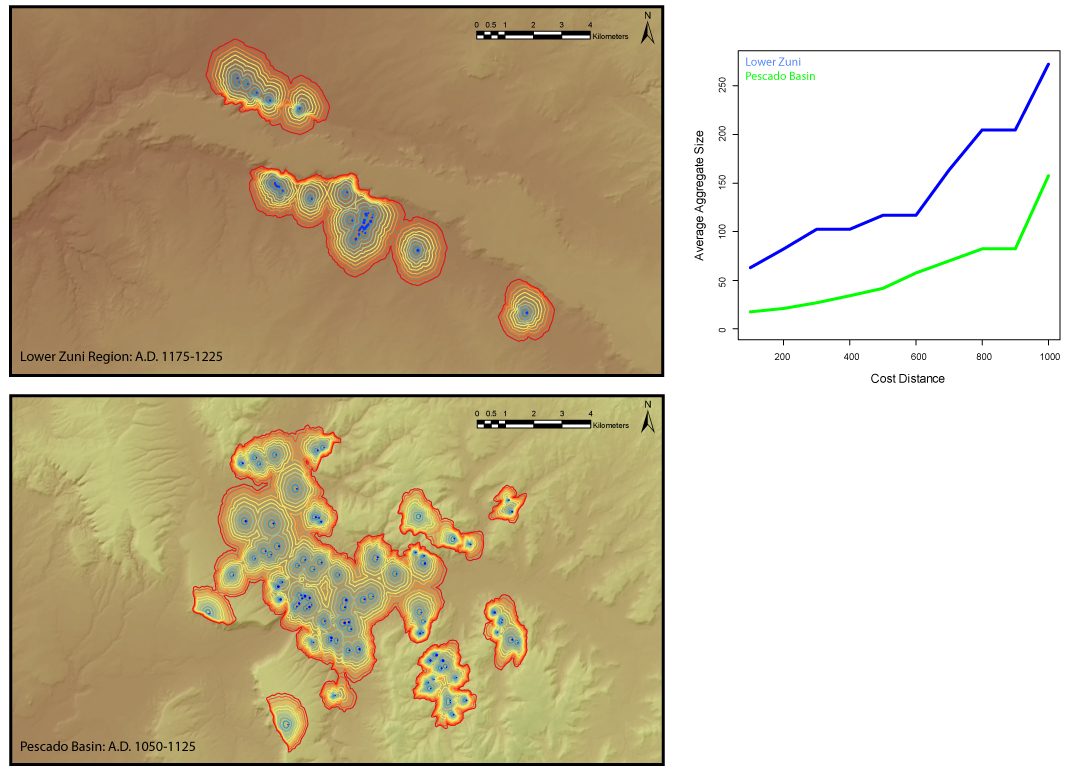


**Fig A.** **Cost Distance Buffers for Two Zuni Locations.**


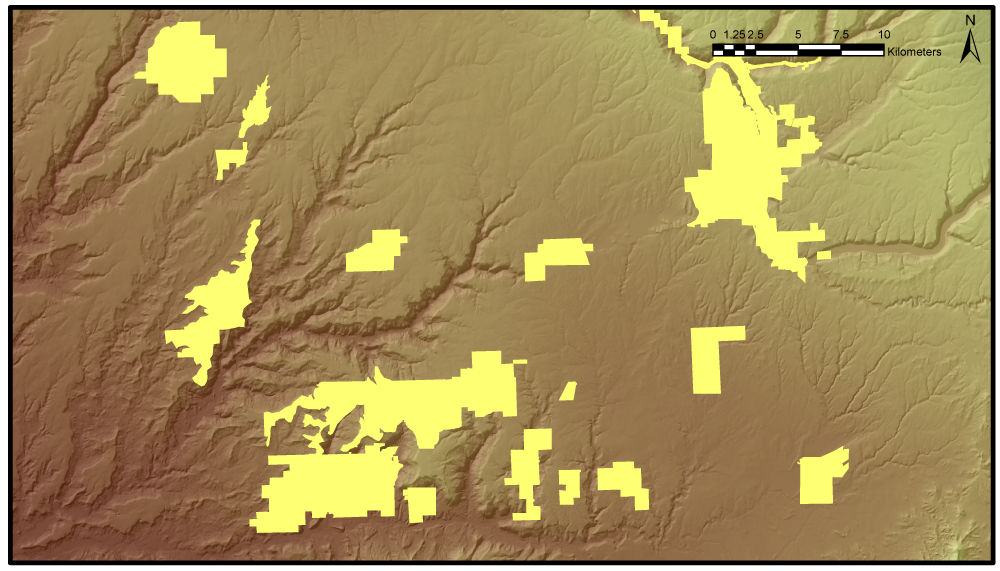


**Fig B.** **Locations of the Large Contiguous Surveys Providing the Data for the Mesa Verde Region.**


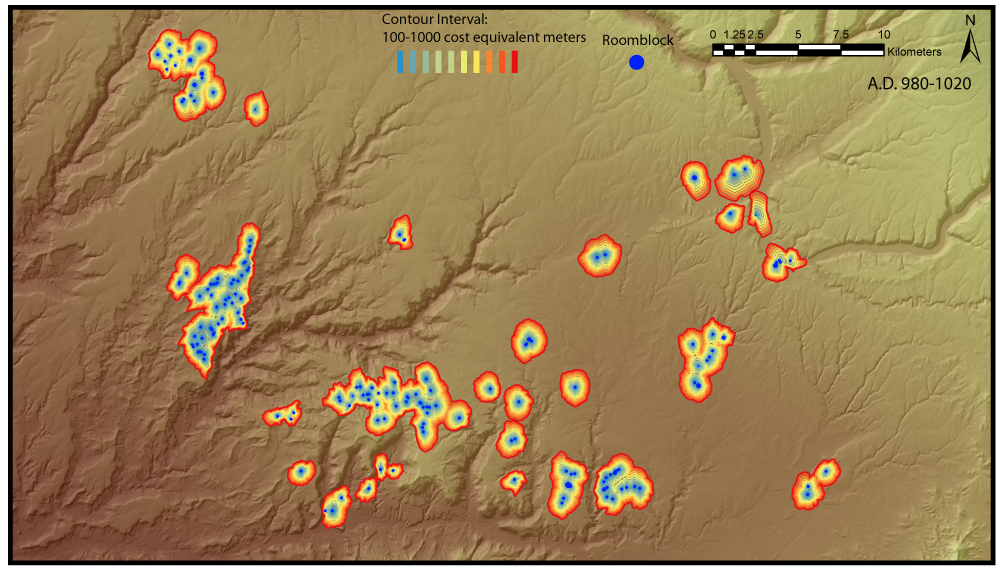


**Fig C.** **The Mesa Verde Region Showing Sites and Cost Equivalent Buffers ca. 980-1020 C.E.**


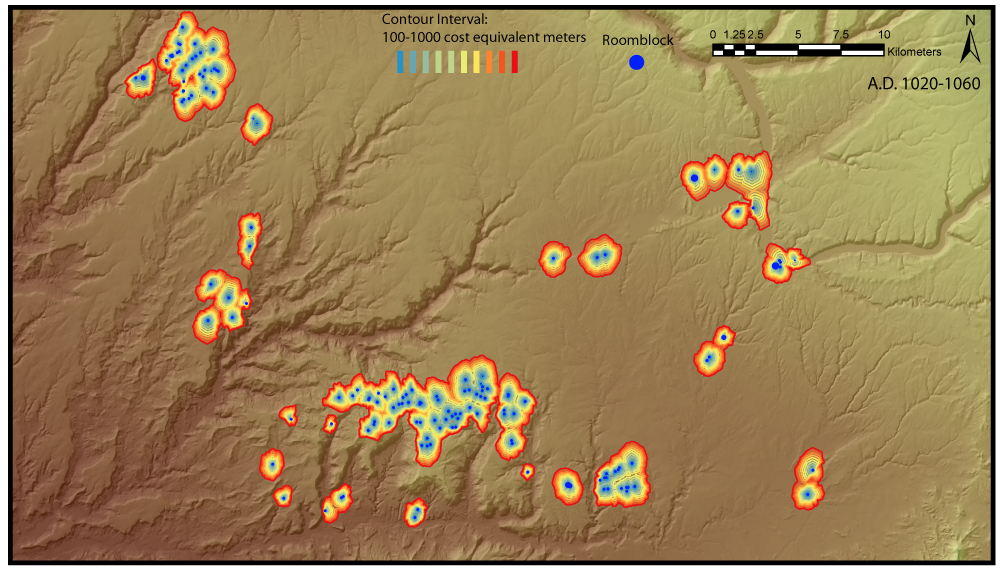


**Fig D.** **The Mesa Verde Region Showing Sites and Cost Equivalent Buffers ca. 1020-1060 C.E.**


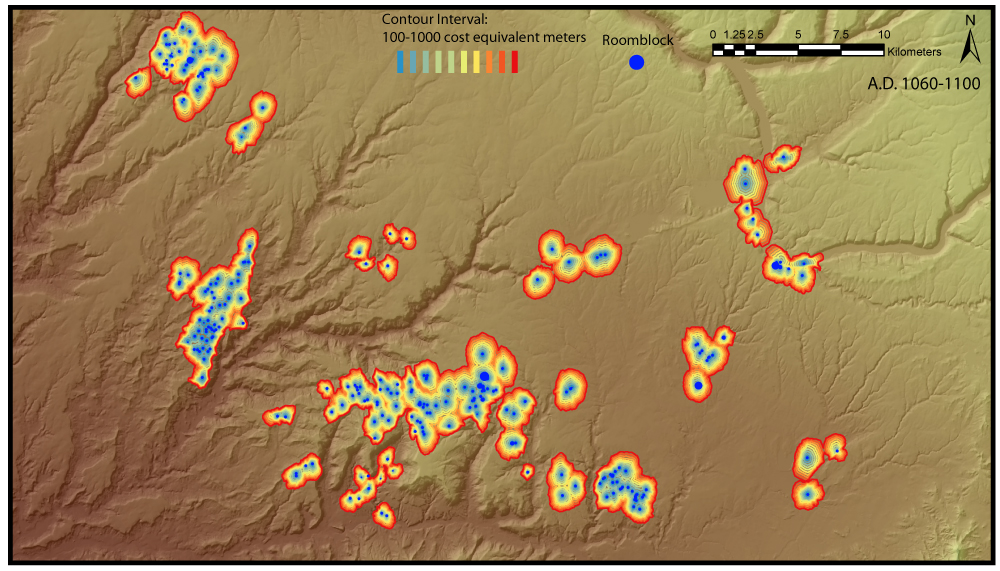


**Fig E.** **The Mesa Verde Region Showing Sites and Cost Equivalent Buffers ca. 1060-1100 C.E.**


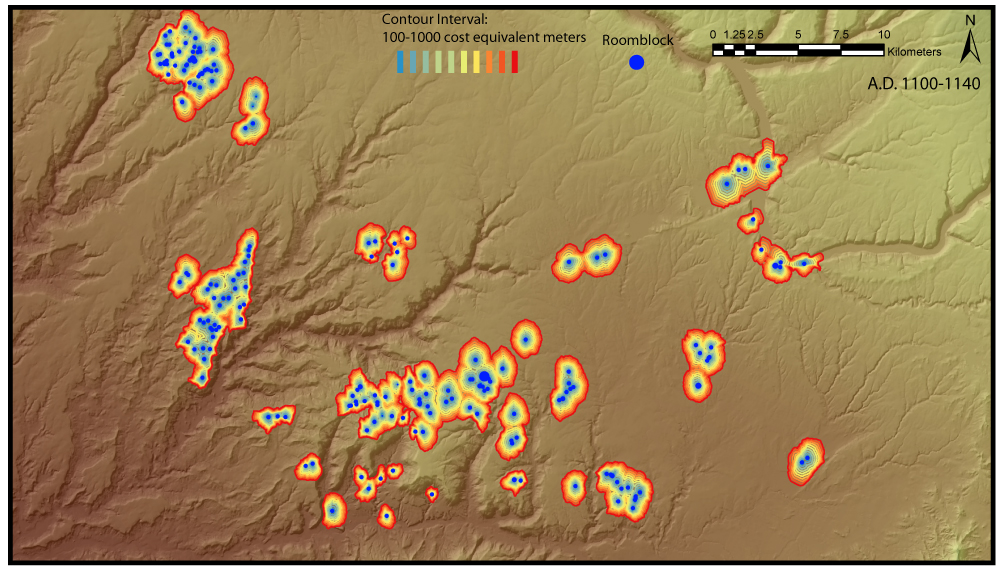


**Fig F.** **The Mesa Verde Region Showing Sites and Cost Equivalent Buffers ca. 1100-1140 C.E.**


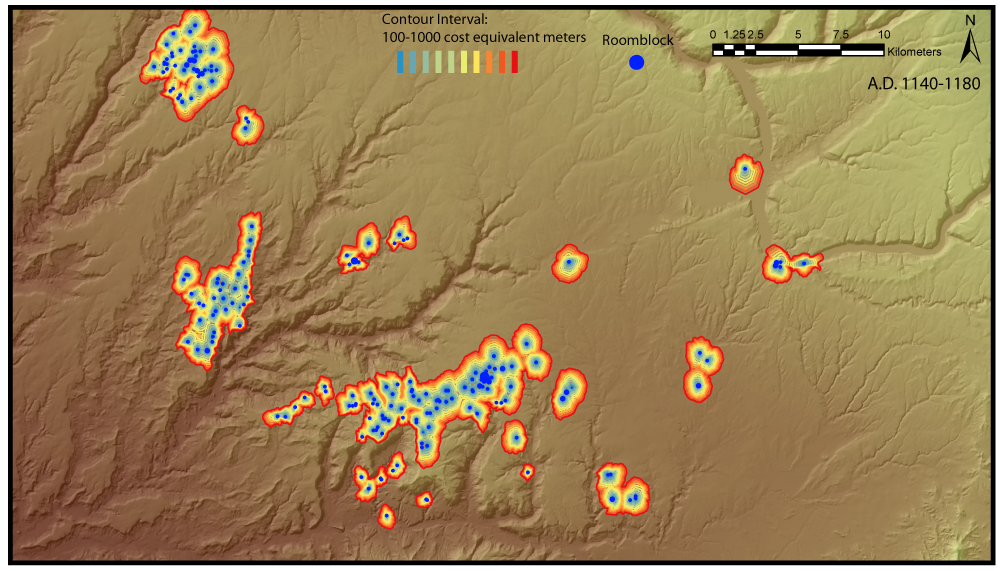


**Fig G.** **The Mesa Verde Region Showing Sites and Cost Equivalent Buffers ca. 1140-1180 C.E.**


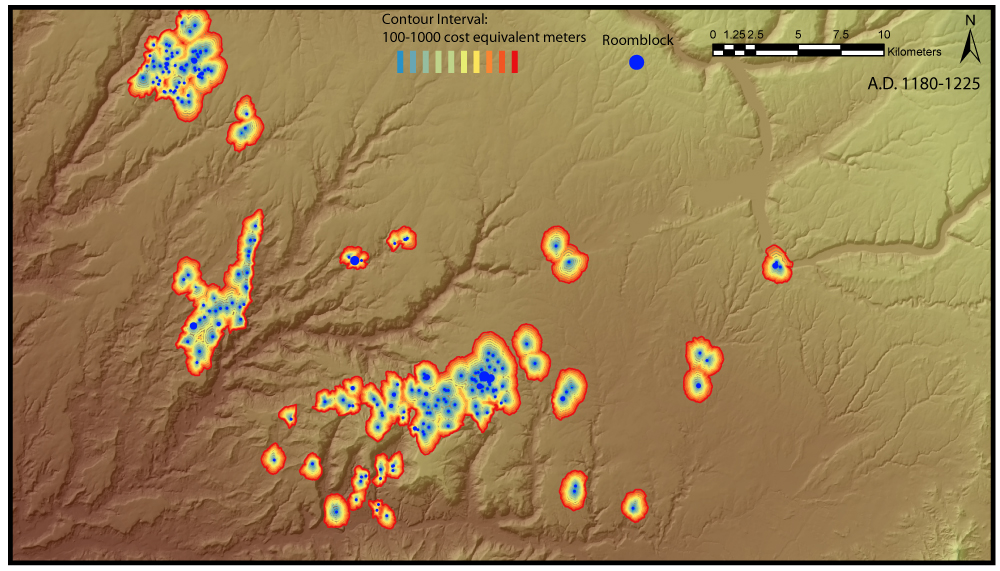


**Fig H.** **The Mesa Verde Region Showing Sites and Cost Equivalent Buffers ca. 1180-1225 C.E.**


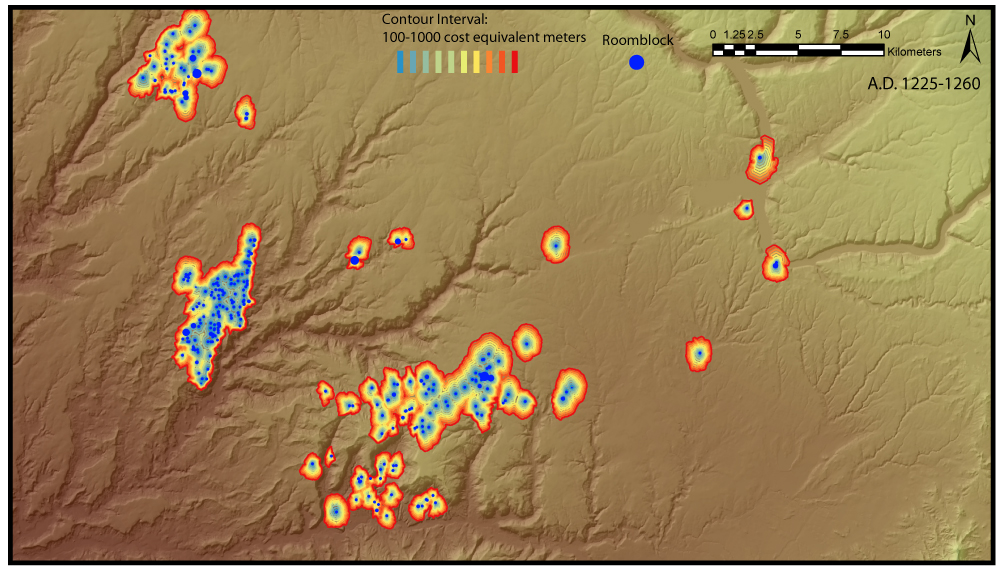


**Fig I.** **The Mesa Verde Region Showing Sites and Cost Equivalent Buffers ca. 1225-1260 C.E.**


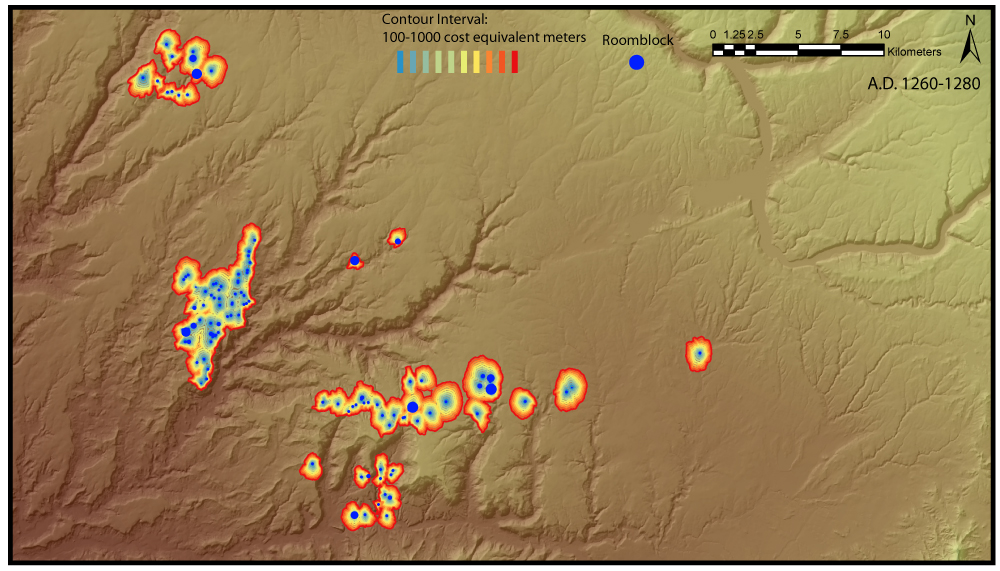


**Fig J.** **The Mesa Verde Region Showing Sites and Cost Equivalent Buffers ca. 1260-1280 C.E.**


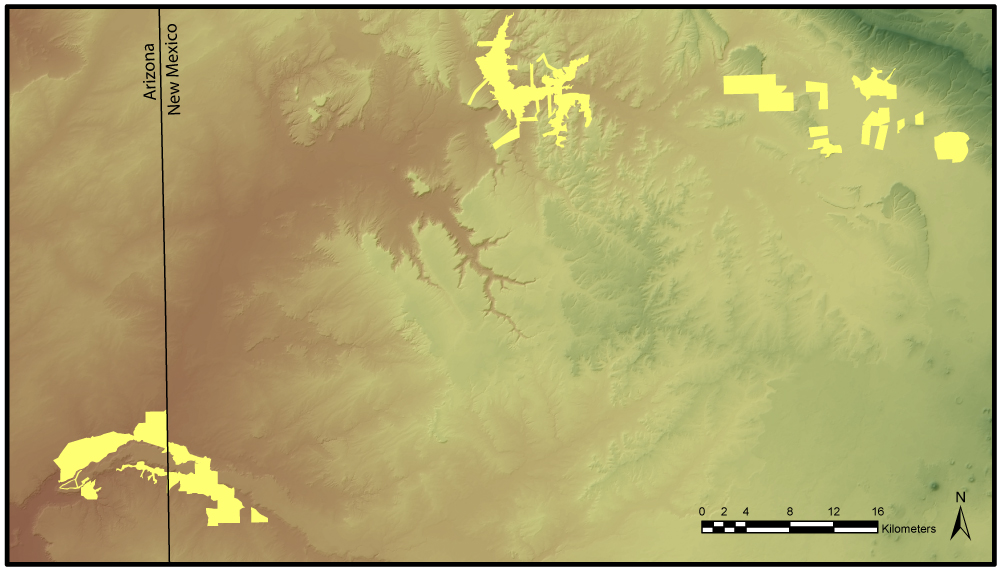


**Fig K.** **Locations of the Large Contiguous Surveys Providing Data for the Zuni Region.**


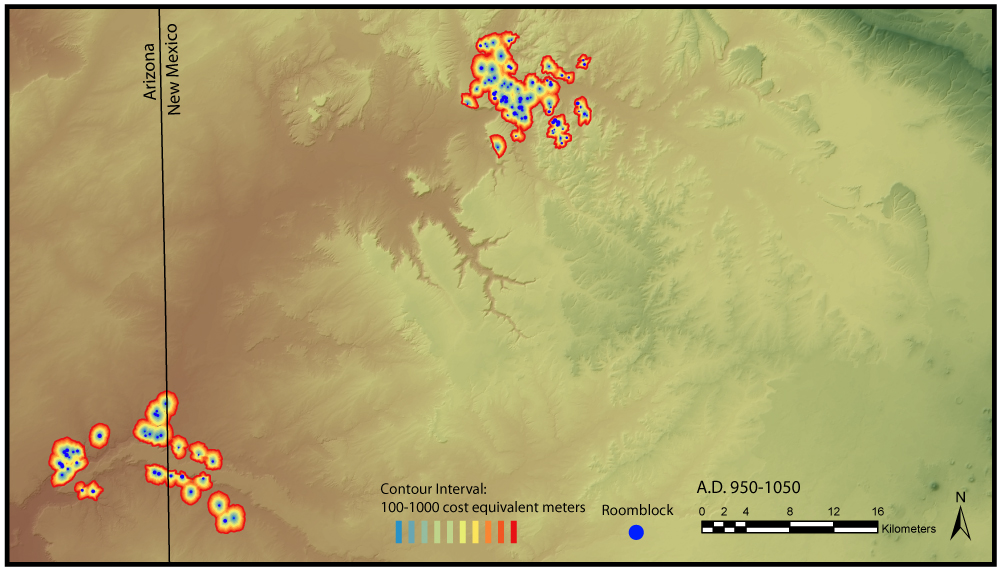


**Fig L**. **The Zuni Region Showing Sites and Cost Equivalent Buffers ca. 950-1050 C.E.**


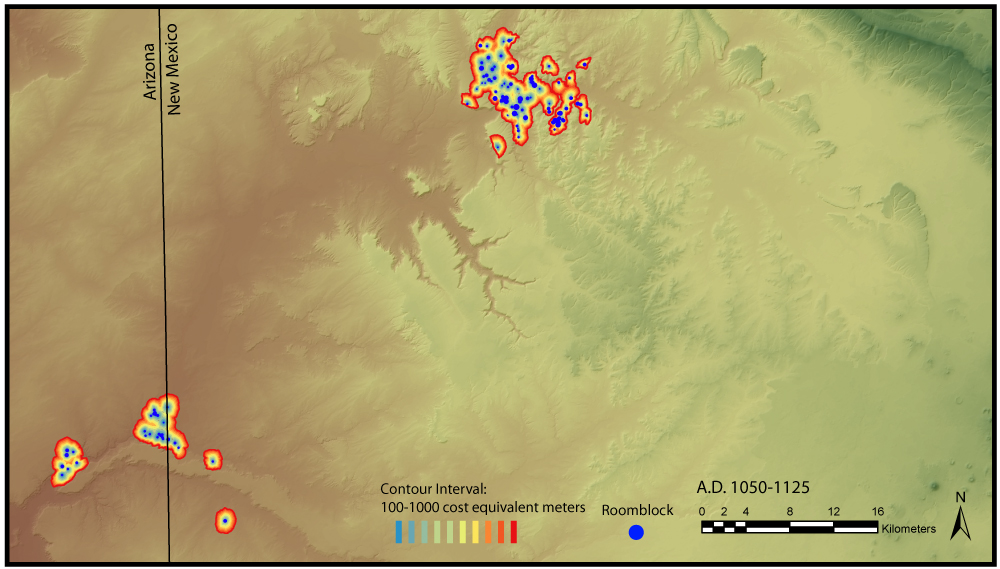


**Fig M**. **The Zuni Region Showing Sites and Cost Equivalent Buffers ca. 1050-1125 C.E.**


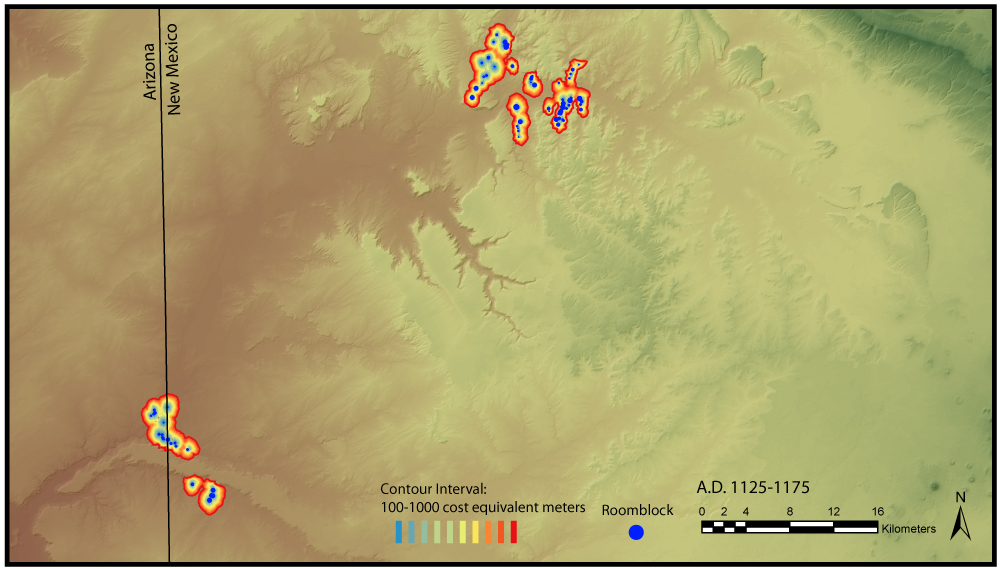


**Fig N**. **The Zuni Region Showing Sites and Cost Equivalent Buffers ca.1125-1175 C.E.**


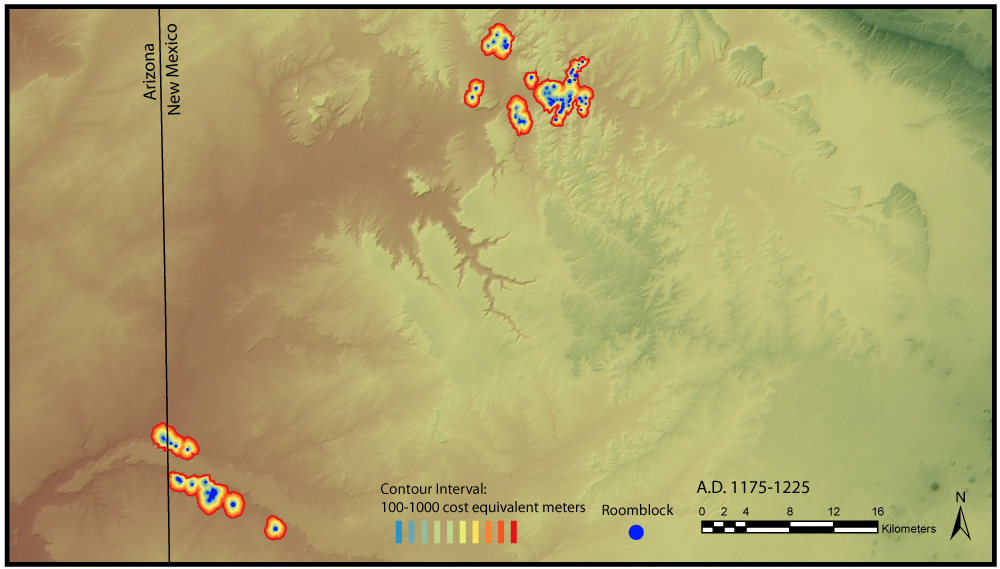


**Fig O**. **The Zuni Region Showing Sites and Cost Equivalent Buffers ca. 1175-1225 C.E.**


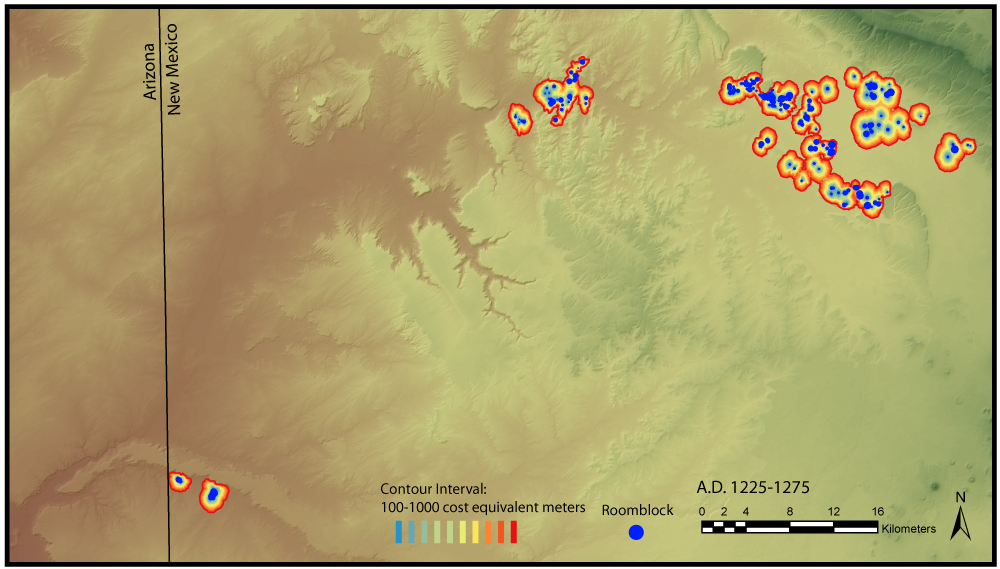


**Fig P**. **The Zuni Region Showing Sites and Cost Equivalent Buffers ca. 1225-1275 C.E.**


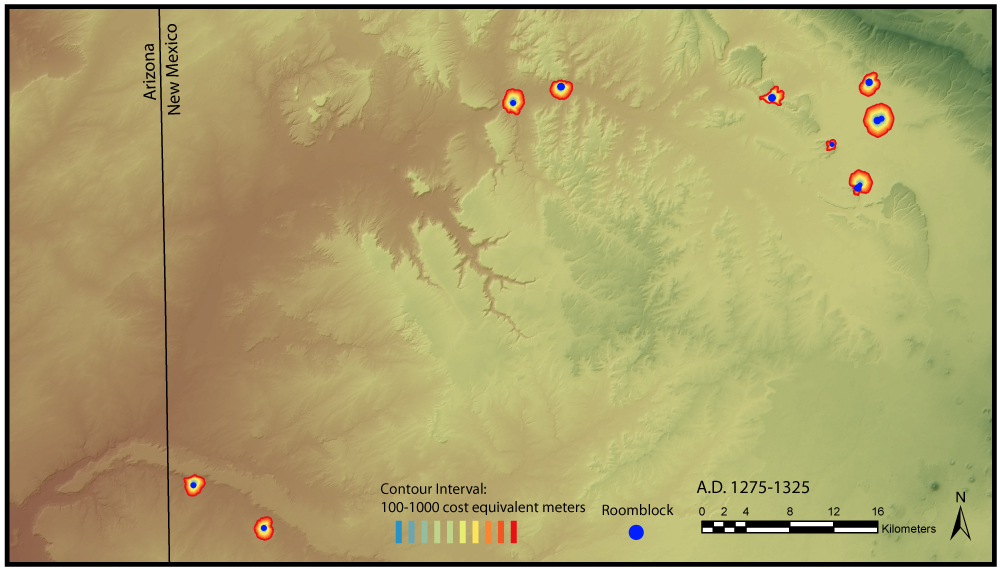


**Fig Q**. **The Zuni Region Showing Sites and Cost Equivalent Buffers ca. 1275-1325 C.E.**


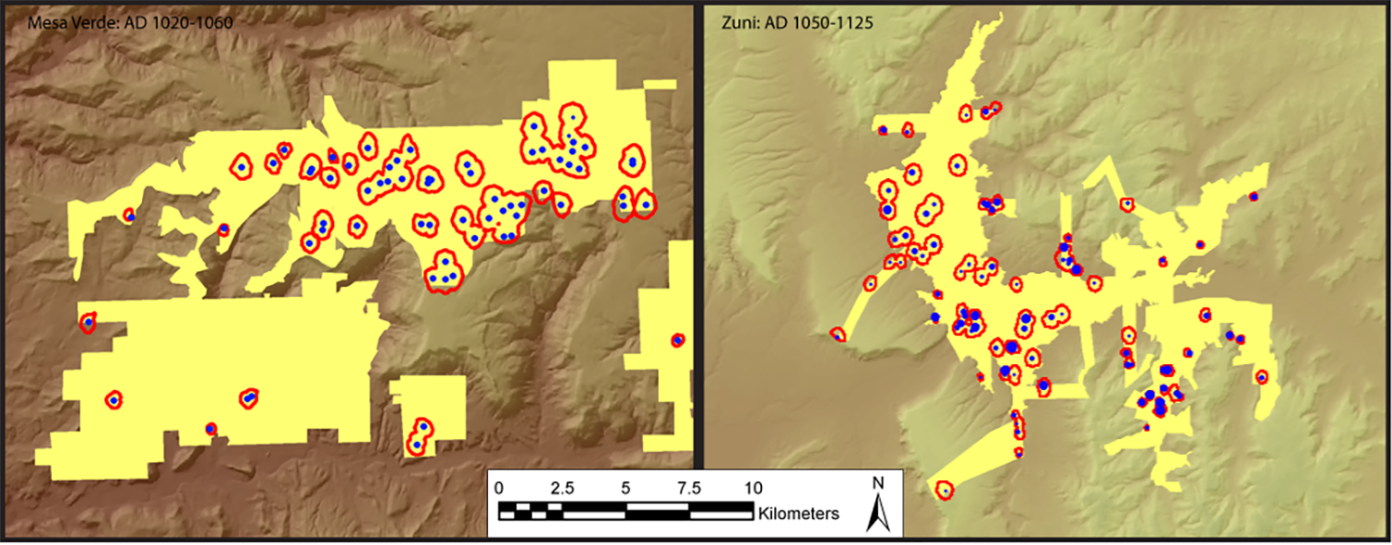


**Fig R.**  **Distributions of Sites in Major Block Surveys in the Mesa Verde and Zuni Regions.** Sites are depicted in blue; the selected cost equivalent distance buffers are shown in red.

**S1 Tables.**

**Table A.** **Summary Statistics for Settlement Size by Period and Case Study.**

| **Zuni Region** |  | | | | | |  |  |
| --- | --- | --- | --- | --- | --- | --- | --- | --- |
|  | **950-1050** | **1050-1125** | **1125-1175** | **1175-1225** | **1225-1275** | **1275-1325** |  |  |
| Mean Aggregate Size (# of rooms) | 13.00 | 16.02 | 25.71 | 45.56 | 61.24 | 607.78 |  |  |
| Range of Aggregate Sizes | 1-99 | 1-150 | 1-171 | 2-568 | 2-1391 | 180-1243 |  |  |
| Standard Deviation of Aggregate Sizes | 15.51 | 22.66 | 34.95 | 91.92 | 162.81 | 426.96 |  |  |
| Coefficient of Variation in Aggregate Size | 1.19 | 1.41 | 1.36 | 2.02 | 2.66 | 0.70 |  |  |
| Skewness of Aggregate Sizes | 3.09 | 3.67 | 2.80 | 4.91 | 6.76 | 0.36 |  |  |
|  |  |  |  |  |  |  |  |  |
|  |  |  |  |  |  |  |  |  |
| **Mesa Verde Region** |  | | | | | | | |
|  | **980-1020** | **1020-1060** | **1060-1100** | **1100-1140** | **1140-1180** | **1180-1225** | **1225-1260** | **1260-1280** |
| Mean Aggregate Size (# of rooms) | 16.11 | 15.00 | 19.15 | 18.07 | 17.75 | 23.56 | 36.55 | 45.05 |
| Range of Aggregate Sizes | 6-102 | 6-66 | 6-324 | 6-318 | 6-354 | 6-588 | 6-906 | 6-606 |
| Standard Deviation of Aggregate Sizes | 18.93 | 14.42 | 40.49 | 37.56 | 41.49 | 68.51 | 119.68 | 112.21 |
| Coefficient of Variation in Aggregate Size | 1.17 | 0.96 | 2.11 | 2.08 | 2.34 | 2.91 | 3.27 | 2.49 |
| Skewness of Aggregate Sizes | 2.58 | 1.88 | 5.62 | 6.30 | 6.51 | 7.17 | 5.87 | 3.91 |

**Table B**. **Public Architectural Information for the Zuni region.** GK = Great Kiva, UGK = Unroofed Great Kiva

| **Site** | **1050-1125** | **1125-1175** | **1175-1225** | **1225-1275** | **1275-1325** | **1325-1375** | **Feature Type** | **GK Diameter** | **Public Arch Area** |
| --- | --- | --- | --- | --- | --- | --- | --- | --- | --- |
| Allentown | 1 | 0 | 0 | 0 | 0 | 0 | GK | 19.2 | 289 |
| Andrews Community Great Kiva 1 | 1 | 0 | 0 | 0 | 0 | 0 | GK | 12 | 113 |
| Atsee Nitsa | 0 | 1 | 1 | 1 | 0 | 0 | UGK | 24 | 452 |
| Box S | 0 | 0 | 0 | 1 | 1 | 0 | UGK | 28 | 615 |
| Casamero Great Kiva 1 | 1 | 0 | 0 | 0 | 0 | 0 | GK | 21 | 346 |
| Casamero Great Kiva 2 | 1 | 0 | 0 | 0 | 0 | 0 | GK | 18 | 254 |
| Cerro Pomo Great Kiva 1 | 1 | 0 | 0 | 0 | 0 | 0 | UGK | 16 | 201 |
| Cerro Pomo Great Kiva 2 | 1 | 0 | 0 | 0 | 0 | 0 | UGK | 16 | 201 |
| Coolidge Great Kiva 1 | 1 | 1 | 0 | 0 | 0 | 0 | GK | 14 | 154 |
| Cox Ranch | 1 | 0 | 0 | 0 | 0 | 0 | UGK | 17 | 227 |
| CS189 | 0 | 0 | 0 | 1 | 1 | 0 | UGK | 30 | 707 |
| Fenced-up Horse Canyon | 0 | 1 | 1 | 1 | 0 | 0 | GK | 18 | 254 |
| Fort Wingate | 1 | 1 | 1 | 0 | 0 | 0 | GK | 12.3 | 119 |
| Garcia Ranch | 0 | 0 | 0 | 1 | 0 | 0 | UGK | 30 | 707 |
| Goesling Ranch | 0 | 1 | 1 | 1 | 0 | 0 | UGK | 22 | 380 |
| Haystack Community Great Kiva 1 | 1 | 0 | 0 | 0 | 0 | 0 | GK | 19 | 283 |
| Haystack Great House | 1 | 0 | 0 | 0 | 0 | 0 | GK | 18 | 254 |
| Hinkson Ranch | 0 | 1 | 1 | 1 | 0 | 0 | UGK | 34 | 907 |
| H-Spear | 1 | 0 | 0 | 0 | 0 | 0 | GK | 14 | 154 |
| Hubble Corner | 0 | 0 | 1 | 1 | 0 | 0 | UGK | 31 | 754 |
| JR Etremitedio | 1 | 0 | 0 | 0 | 0 | 0 | GK | 20 | 314 |
| Kin Cheops I | 1 | 0 | 0 | 0 | 0 | 0 | UGK | 20 | 314 |
| Kin HochoI Great Kiva 2 | 1 | 1 | 1 | 0 | 0 | 0 | GK | 22 | 380 |
| Kluckhohn | 0 | 0 | 0 | 1 | 1 | 0 | UGK | 34 | 907 |
| Las Ventanas | 1 | 1 | 1 | 0 | 0 | 0 | GK | 17 | 227 |
| Los Gigantes | 0 | 0 | 0 | 1 | 0 | 0 | UGK | 31 | 754 |
| Los Veteados | 1 | 0 | 0 | 0 | 0 | 0 | GK | 22 | 380 |
| McCreery | 1 | 1 | 1 | 0 | 0 | 0 | UGK | 18 | 254 |
| Navajo Springs | 1 | 0 | 0 | 0 | 0 | 0 | GK | 20 | 314 |
| Skull Site | 1 | 0 | 0 | 0 | 0 | 0 | GK | 13 | 133 |
| Spier 81 | 0 | 0 | 1 | 0 | 0 | 0 | GK | 14 | 154 |
| Vidal Canyon | 1 | 0 | 0 | 0 | 0 | 0 | GK | 16.1 | 203 |
| Village of the Great Kivas 1 | 1 | 1 | 1 | 0 | 0 | 0 | GK | 16.6 | 216 |
| Village of the Great Kivas 2 | 0 | 1 | 1 | 0 | 0 | 0 | UGK | 23.8 | 445 |
| Atsinna | 0 | 0 | 0 | 0 | 1 | 1 | Plaza | NA | 2440 |
| North Atsinna | 0 | 0 | 0 | 0 | 1 | 0 | Plaza | NA | 870 |
| Pueblo de los Muertos | 0 | 0 | 0 | 0 | 1 | 1 | Plaza | NA | 3685 |
| Ramah School | 0 | 0 | 0 | 0 | 1 | 0 | Plaza | NA | 1140 |
| Lower Deracho Ruin | 0 | 0 | 0 | 1 | 0 | 0 | Plaza | NA | 555 |
| Heshotauthla | 0 | 0 | 0 | 0 | 1 | 1 | Plaza | NA | 3725 |
| Yellowhouse | 0 | 0 | 0 | 0 | 1 | 1 | Plaza | NA | 1740 |
| Lower Pescado Ruin | 0 | 0 | 0 | 0 | 0 | 1 | Plaza | NA | 1730 |
| Archeotekopa II | 0 | 0 | 0 | 1 | 1 | 0 | Plaza | NA | 2680 |
| Fort Site | 0 | 0 | 0 | 1 | 1 | 0 | Plaza | NA | 1518 |
| Horse Camp Mill | 0 | 0 | 0 | 1 | 1 | 0 | Plaza | NA | 3850 |
| Veteado | 0 | 0 | 0 | 1 | 1 | 0 | Plaza | NA | 4880 |
| Rattail | 0 | 0 | 0 | 1 | 1 | 0 | Plaza | NA | 1200 |
| Los Pilares | 0 | 0 | 0 | 1 | 1 | 0 | Plaza | NA | 4059 |

**Table C. Public Architectural Information for 24 of the Late, Aggregated Villages in the VEP I study area within the Mesa Verde.** GK = Great Kiva. Note that in some cases sites are listed twice if they have both a great kiva and a plaza.

| **Site** | **1060-1140** | **1140-1180** | **1180-1225** | **1225-1260** | **1260-1280** | **Feature Type** | **Public Arch Area** |
| --- | --- | --- | --- | --- | --- | --- | --- |
| 5MT107 | 1 | 1 | 0 | 0 | 0 | GK | 210 |
| Hartman Draw | 1 | 1 | 1 | 0 | 0 | GK | 331 |
| Lower Cow Canyon | 0 | 0 | 0 | 1 | 1 | GK | 80 |
| Kristie's Site | 0 | 1 | 1 | 1 | 1 | GK | 183 |
| Hampton (Squaw Point) Ruin | 1 | 1 | 1 | 1 | 1 | GK | 82 |
| Yellow Jacket Pueblo | 1 | 1 | 1 | 1 | 1 | GK | 91 |
| Yucca House | 0 | 1 | 1 | 1 | 1 | GK | 248 |
| Brewer Canyon Pueblo | 0 | 0 | 1 | 1 | 1 | GK | 134 |
| Kearns' Site | 0 | 0 | 1 | 1 | 1 | GK | 80 |
| Sand Canyon Pueblo | 0 | 0 | 0 | 0 | 1 | GK | 154 |
| Seven Towers Pueblo | 0 | 0 | 0 | 1 | 1 | GK | 58 |
| Goodman Point Pueblo | 0 | 0 | 0 | 0 | 1 | GK | 187 |
| Hartman Draw | 1 | 1 | 1 | 0 | 0 | Plaza | 444 |
| Lower Cow Canyon | 0 | 0 | 0 | 1 | 1 | Plaza | 42 |
| Big Spring Ruin | 0 | 0 | 0 | 1 | 1 | Plaza | 70 |
| Bear Paw Pueblo | 0 | 0 | 0 | 1 | 1 | Plaza | 58 |
| Painted Hand Pueblo | 0 | 0 | 0 | 1 | 1 | Plaza | 99 |
| Cow Mesa 40 | 0 | 0 | 1 | 1 | 1 | Plaza | 28 |
| Mud Springs | 0 | 1 | 1 | 1 | 1 | Plaza | 4405 |
| Turkey House Complex | 0 | 0 | 0 | 1 | 1 | Plaza | 37 |
| Woods Canyon Pueblo | 0 | 1 | 1 | 1 | 1 | Plaza | 684 |
| Thompson Site | 0 | 0 | 1 | 1 | 1 | Plaza | 268 |
| Cannonball Ruins | 0 | 0 | 0 | 1 | 1 | Plaza | 280 |
| Yellow Jacket Pueblo | 1 | 1 | 1 | 1 | 1 | Plaza | 14814 |
| Little Cow Canyon | 0 | 0 | 0 | 1 | 1 | Plaza | 52 |
| Yucca House | 0 | 1 | 1 | 1 | 1 | Plaza | 4666 |
| Brewer Canyon Pueblo | 0 | 0 | 1 | 1 | 1 | Plaza | 1068 |
| Sand Canyon Pueblo | 0 | 0 | 0 | 0 | 1 | Plaza | 841 |
| Seven Towers Pueblo | 0 | 0 | 0 | 1 | 1 | Plaza | 193 |
| Castle Rock Pueblo | 0 | 0 | 0 | 0 | 1 | Plaza | 488 |
| Pedro Point | 0 | 0 | 0 | 1 | 1 | Plaza | 352 |
| Goodman Point Pueblo | 0 | 0 | 0 | 0 | 1 | Plaza | 1928 |
